# SIRT7 antagonizes human stem cell aging as a heterochromatin stabilizer

**DOI:** 10.1007/s13238-020-00728-4

**Published:** 2020-06-06

**Authors:** Shijia Bi, Zunpeng Liu, Zeming Wu, Zehua Wang, Xiaoqian Liu, Si Wang, Jie Ren, Yan Yao, Weiqi Zhang, Moshi Song, Guang-Hui Liu, Jing Qu

**Affiliations:** 1 State Key Laboratory of Stem Cell and Reproductive Biology, Institute of Zoology, Chinese Academy of Sciences, Beijing, 100101 China; 2 State Key Laboratory of Membrane Biology, Institute of Zoology, Chinese Academy of Sciences, Beijing, 100101 China; 3 Beijing Institute for Brain Disorders, Advanced Innovation Center for Human Brain Protection, National Clinical Research Center for Geriatric Disorders, Xuanwu Hospital Capital Medical University, Beijing, 100053 China; 4 CAS Key Laboratory of Genomic and Precision Medicine, Beijing Institute of Genomics, Chinese Academy of Sciences, Beijing, 100101 China; 5 China National Center for Bioinformation, Beijing, 100101 China; 6 Institute for Stem cell and Regeneration, Chinese Academy of Sciences, Beijing, 100101 China; 7 University of Chinese Academy of Sciences, Beijing, 100049 China; 8 Department of Cardiology, Beijing Anzhen Hospital, Capital Medical University, Beijing, 100029 China

**Keywords:** SIRT7, stem cell, aging, LINE1, cGAS, STING

## Abstract

SIRT7, a sirtuin family member implicated in aging and disease, is a regulator of metabolism and stress responses. It remains elusive how human somatic stem cell populations might be impacted by SIRT7. Here, we found that SIRT7 expression declines during human mesenchymal stem cell (hMSC) aging and that SIRT7 deficiency accelerates senescence. Mechanistically, SIRT7 forms a complex with nuclear lamina proteins and heterochromatin proteins, thus maintaining the repressive state of heterochromatin at nuclear periphery. Accordingly, deficiency of SIRT7 results in loss of heterochromatin, de-repression of the LINE1 retrotransposon (LINE1), and activation of innate immune signaling via the cGAS-STING pathway. These aging-associated cellular defects were reversed by overexpression of heterochromatin proteins or treatment with a LINE1 targeted reverse-transcriptase inhibitor. Together, these findings highlight how SIRT7 safeguards chromatin architecture to control innate immune regulation and ensure geroprotection during stem cell aging.

**Electronic supplementary material:**

The online version of this article (10.1007/s13238-020-00728-4) contains supplementary material, which is available to authorized users.

## Introduction

Sirtuins, mammalian homologs of the yeast longevity protein Sir2, are evolutionarily conserved nicotinamide adenine dinucleotide (NAD^+^)-dependent histone deacetylases (HDACs) involved in metabolic regulation and aging (Bishop and Guarente, 2007; Finkel et al., 2009; Herskovits and Guarente, 2013; Zhang et al., 2018). SIRT7 is the only sirtuin member that mainly localizes to the nucleolus (Michishita et al., 2005), a site for ribosome biogenesis and stress response (Shin et al., 2013; Tsai et al., 2014; Chen et al., 2016). Previous studies show that Sirt7-deficient mice have a shortened lifespan and suffer from aging-associated disorders (Vakhrusheva et al., 2008; Vazquez et al., 2016; Wronska et al., 2016). At the physiological level, SIRT7 has been implicated in hepatic lipid metabolism, cardiovascular system homeostasis and adipogenesis (Yoshizawa et al., 2014; Araki et al., 2015; Cioffi et al., 2015). At molecular levels, SIRT7 is broadly recognized for safeguarding genome integrity (Kiran et al., 2015; Vazquez et al., 2016; Paredes et al., 2018; Bao et al., 2019). For example, SIRT7 targets both acetylated H3 lysine 18 (Barber et al., 2012) and succinylated H3 lysine 122 (Li et al., 2016), and is recruited to DNA double-strand breaks (DSBs) in a PARP1-dependent manner, promoting chromatin condensation and DSB repair (Vazquez et al., 2016). Besides, SIRT7 is known as a direct transcriptional repressor that regulates mitochondrial and cytosolic protein homeostasis (Barber et al., 2012; Shin et al., 2013; Mohrin et al., 2015). Despite many seminal advances furthering our understanding of SIRT7 biology, the identification of reversible SIRT7-mediated cellular aging processes remains a challenge for the field.

Aging is a complicated process characterized by a progressive reduction in physiological integrity and organ function (Liu et al., 2012; Singh et al., 2019; He et al., 2020). A major contributor to organismal aging is stem cell senescence and exhaustion, which disrupts tissue maintenance and impairs organ regeneration (Ren et al., 2017). Human mesenchymal stem cells (hMSCs) support the maintenance of other cell populations in the body and have the potential to differentiate into diverse cell lineages, such as chondrocytes, osteoblasts, and adipocytes (Uccelli et al., 2008; Dimarino et al., 2013; Obeid et al., 2013). Not entirely surprisingly, progressive exhaustion of the hMSC pool has been causally linked to aging-associated tissue malfunction and degenerative diseases (Zhou et al., 2008; Zhang et al., 2011; Yang, 2018). In premature aging diseases like Hutchinson-Gilford progeria syndrome (HGPS), the hMSC population also undergoes accelerated decay with features of classic cellular senescence (Stenderup et al., 2003; Kudlow et al., 2007; Liu et al., 2011; Kubben et al., 2016; Wu et al., 2018). Small-molecule chemicals with geroprotective activity on hMSCs have exhibited efficacy to prolong healthspan and alleviate aging-associated syndromes in rodents (Geng et al., 2019). Thus, exploring molecular mechanisms of hMSC senescence can provide us with insight into how to ameliorate the development of age-related diseases or reverse cellular aging.

Cellular senescence is accompanied by marked epigenetic changes including altered DNA methylation patterns, aberrant histone modifications, and misleading nucleosome positioning (Talens et al., 2012; Lopez-Otin et al., 2013; Ren et al., 2017; Zhang et al., 2020). Heterochromatin disorganization, in particular, has been considered as a driver contributing to hMSC senescence in aging and diseases (Zhang et al., 2015; Deng et al., 2019). Heterochromatin endows particular genomic domains with enrichment of histone H3 Lys9 trimethylation (H3K9me3) and heterochromatin protein 1 α (HP1α) (Sridharan et al., 2013; Grewal and Jia, 2007; Kubben and Misteli, 2017). Such condensed heterochromatin prevents unequal recombination between repetitive regions and confers repression of transposable elements (TE) (Bourque et al., 2018; Zhang et al., 2020) that are primarily located at genomic lamina-associated domains (LADs) and spatially anchored to the nuclear periphery with nuclear lamina proteins (Guelen et al., 2008; van Steensel and Dekker, 2010; Bickmore and van Steensel, 2013). Emerging shreds of evidence have indicated that de-repression of retrotransposons such as long interspersed element 1 (LINE1) occurs during organismal and cellular aging and could be a new driver for cellular senescence (Garcia-Perez et al., 2010; Gorbunova et al., 2014; Van Meter et al., 2014; De Cecco et al., 2019; Simon et al., 2019). To date, the mechanisms underlining heterochromatin maintenance in human stem cells are largely unknown.

In this study, we discovered that SIRT7 functions to tether heterochromatin to the nuclear lamina and thereby maintains heterochromatin organization in hMSCs. We also found that SIRT7 downregulation associated with hMSC senescence accounts for detachment of LADs from the nuclear lamina, loss of heterochromatin, and de-repression of LINE1, events that activate the innate immune response through the cyclic GMP-AMP synthase-stimulator of interferon genes (cGAS-STING) signaling. Restoration of heterochromatin compaction or inhibition of LINE1 retrotransposition rescued the senescence phenotypes in SIRT7-deficient hMSCs. These results reveal that leveraging heterochromatin-innate immune response pathways is a novel facet by which SIRT7 safeguards hMSC integrity.

## Results

### SIRT7 is downregulated in senescent hMSCs

**Figure 1:**
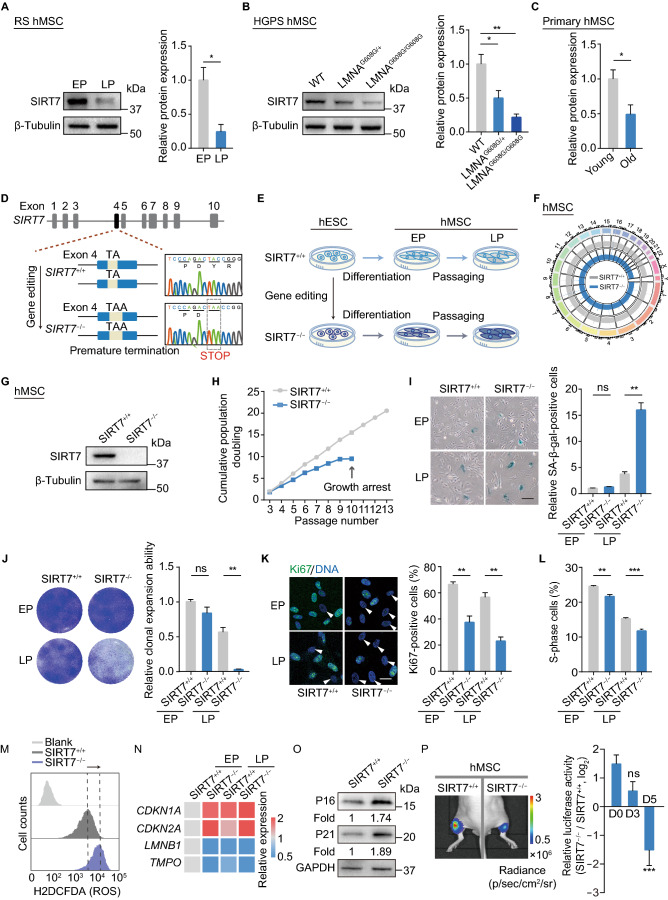
**Generation and characterization of SIRT7-deficient hESCs**. (A) Left, Western blot analysis of SIRT7 protein in replicatively senescent (RS) hMSCs at early (EP, P3) and late passages (LP, P8) with β-Tubulin as loading control. Right, statistical analysis of relative SIRT7 protein expression levels. Data are presented as the means ± SEM. *n* = 3. *, *P* < 0.05 (*t* test). (B) Left, Western blot analysis of SIRT7 protein in WT and HGPS-specific (*LMNA*^G608G/+^ or *LMNA*^G608G/G608G^) hMSCs at LP (P8) with β-Tubulin used as loading control. Right, statistical analysis of relative SIRT7 protein expression levels. Data are presented as the means ± SEM. *n* = 3. *, *P* < 0.05, **, *P* < 0.01 (*t* test). (C) Statistical analysis of relative SIRT7 protein expression levels in young and old primary hMSCs. Data are presented as the means ± SEM. *n* = 4 samples. *, *P* < 0.05 (*t* test). (D) Left, schematic illustration of *SIRT7* gene editing (exon 4) using CRISPR/Cas9-mediated non-homologous end joining (NHEJ) in hESCs. Right, DNA sequence chromatogram showing the introduction of termination codon TAA by gene editing. (E) Schematic workflow showing the generation of *SIRT7*^+/+^ and *SIRT7*^−/−^ hMSCs from hESCs. (F) Genome-wide analysis of copy number variations (CNVs) in *SIRT7*^+/+^ and *SIRT7*^−/−^ hMSCs at middle passage (MP, P6). (G) Western blot analysis of SIRT7 protein in *SIRT7*^+/+^ and *SIRT7*^−/−^ hMSCs with β-Tubulin used as loading control. (H) Growth curves of *SIRT7*^+/+^ and *SIRT7*^−/−^ hMSCs. Data are presented as the means ± SEM. *n* = 3. (I) SA-β-gal staining of *SIRT7*^+/+^ and *SIRT7*^−/−^ hMSCs at EP (P3) and LP (P8). Scale bar, 125 μm. Data are presented as the means ± SEM. *n* = 3. ns, not significant, **, *P* < 0.01 (*t* test). (J) Clonal expansion analysis of *SIRT7*^+/+^ and *SIRT7*^−/−^ hMSCs at EP (P3) and LP (P8). Data are presented as the means ± SEM. *n* = 3. ns, not significant, **, *P* < 0.01 (*t* test). (K) Immunostaining of Ki67 in *SIRT7*^+/+^ and *SIRT7*^−/−^ hMSCs at EP (P3) and LP (P8). Scale bar, 25 μm. Data are presented as the means ± SEM. *n* = 3. **, *P* < 0.01 (*t* test). (L) Bar plot showing the percentages of cells in S-phase of cell cycle in *SIRT7*^+/+^ and *SIRT7*^−/−^ hMSCs at EP (P3) and LP (P8). Data are presented as the means ± SEM. *n* = 3. **, *P* < 0.01, ***, *P* < 0.001 (*t* test). (M) ROS levels were determined by staining with the free radical sensor H2DCFDA and measured by FACS in *SIRT7*^+/+^ and *SIRT7*^−/−^ hMSCs at MP (P6). Data are presented as the means ± SEM. *n* = 3. (N) Heatmap showing quantitative RT-PCR analysis of aging-related genes in *SIRT7*^+/+^ and *SIRT7*^−/−^ hMSCs at EP (P3) and LP (P8) hMSCs. Expression levels of the indicated genes in each cell type were normalized to those in *SIRT7*^+/+^ hMSCs at EP (P3). (O) Western blot analysis of cyclin-dependent kinase inhibitors P16 and P21 proteins in *SIRT7*^+/+^ and *SIRT7*^−/−^ hMSCs at MP (P6) with GAPDH used as loading control. (P) Analysis of luciferase activities in TA muscles of immunodeficient mice transplanted with *SIRT7*^+/+^ (left) or *SIRT7*^−/−^ hMSCs (right) at MP (P6) in Day 0, 3 and 5 after implantation. Data calculated by the ratios of log_2_ (*SIRT7*^−/−^/*SIRT7*^+/+^) are presented as the means ± SEM. *n* = 6. ns, not significant, ***, *P* < 0.001 (*t* test)

To evaluate SIRT7 expression in human cellular models of aging, we first measured the SIRT7 protein level in replicatively senescent (RS) hMSCs and HGPS-specific (*LMNA*^G608G/+^ or *LMNA*^G608G/G608G^) hMSCs, known to be senescent prematurely (Liu et al., 2011; Wu et al., 2018). Western blot analysis revealed that SIRT7 protein level was decreased in late-passage versus early-passage wild-type (WT, *SIRT7*^+/+^) hMSCs but also in HGPS hMSCs relative to control hMSCs (Fig. 1A and 1B). We also found that SIRT7 was downregulated in primary hMSCs derived from physiologically aged individuals relative to hMSCs derived from younger individuals (Figs. 1C and S1A). Collectively, these data suggest that the downregulation of SIRT7 is a phenotype of cellular aging in hMSCs.

### SIRT7 deficiency accelerates hMSC senescence

Using a CRISPR/Cas9-assisted gene knockout strategy with sgRNAs targeting *SIRT7*, we next generated SIRT7-deficient human embryonic stem cells (hESCs), which were homozygous for a mutation in *SIRT7* resulting in premature termination of SIRT7 translation (Fig. 1D). Successful ablation of SIRT7 protein was verified with Western blot (Fig. S1B) while karyotyping and genome-wide copy number variation (CNV) analyses demonstrated that the genomic integrity of SIRT7-deficient (*SIRT7*^−/−^) hESCs remained intact (Fig. S1C and S1D). Also, we observed normal morphology and comparable expression of pluripotency markers NANOG, SOX2 and OCT4 in *SIRT7*^−/−^ hESCs relative to *SIRT7*^+/+^ hESCs (Fig. S1E). No difference in proliferative potential was observed between *SIRT7*^+/+^ and *SIRT7*^−/−^ hESCs (Fig. S1F). In all, these data suggest that SIRT7 is dispensable for maintaining hESC properties.

We then differentiated *SIRT7*^+/+^ and *SIRT7*^−/−^ hESCs into hMSCs (referred to hereafter as *SIRT7*^+/+^ and *SIRT7*^−/−^ hMSCs, respectively) (Fig. 1E). Both *SIRT7*^+/+^ and *SIRT7*^−/−^ hMSCs were positive for typical hMSC surface markers including CD105, CD90, CD73, CD44, CD166 and CD29, and negative for non-hMSC markers, such as CD164, CD14, CD19 and podoplanin (PDPN) (Fig. S2A) (Secunda et al., 2015; Miura, 2016). Both types of hMSCs maintained chromosomal integrity as verified with CNV analysis (Fig. 1F), and the absence of SIRT7 protein (Fig. 1G) had no noticeable effect on hMSC differentiation into osteoblasts, chondrocytes, and adipocytes (Fig. S2B).

To investigate whether SIRT7 deficiency accelerates hMSC senescence, we subjected *SIRT7*^+/+^ and *SIRT7*^−/−^ hMSCs to serial passaging. Compared with *SIRT7*^+/+^ hMSCs, which exhibited normal aging kinetics, *SIRT7*^−/−^ hMSCs underwent complete growth arrest as early as by passage 10 (Fig. 1H). We also observed increased percentages of senescence-associated β-galactosidase (SA-β-gal)-positive cells (Fig. 1I), compromised clonal expansion ability (Fig. 1J), decreased ratios of Ki67-positive cells (Fig. 1K) and reduced percentages of S-phase cells in *SIRT7*^−/−^ relative to *SIRT7*^+/+^ hMSCs (Fig. 1L). In addition, SIRT7 deficiency resulted in higher reactive oxygen species (ROS) levels and an increased DNA damage response (DDR) (Figs. 1M, S2C and S2D). Consistent with the accelerated senescence phenotypes, we observed the upregulation of cyclin-dependent kinase inhibitors and aging markers *CDKN1A* (P21) and *CDKN2A* (P16) at both mRNA and protein levels, along with transcriptional downregulation of *LMNB1* and *TMPO* in *SIRT7*^−/−^ hMSCs (Fig. 1N and 1O). RNA sequencing (RNA-seq) results further showed that genes differentially expressed between *SIRT7*^−/−^ and *SIRT7*^+/+^ hMSCs were primarily associated with the terms “aging” and “senescence-associated secretory phenotype (SASP)” (Fig. S2E–G). Finally, *SIRT7*^−/−^ hMSCs underwent accelerated decay *in vivo* when implanted into the tibialis anterior (TA) muscles of immunodeficient mice relative to *SIRT7*^+/+^ hMSCs (Fig. 1P).

To confirm a direct role for SIRT7 in hMSC senescence, we transduced WT hMSCs with lentiviruses encoding CRISPR/Cas9 and *SIRT7*-targeted sgRNAs (Sanjana et al., 2014) (Fig. S2H). Similar to what we had observed in SIRT7-null hESC-derived hMSCs, SIRT7 ablation resulted in increased percentages of SA-β-gal-positive cells and decreased clonal expansion ability, reflecting accelerated senescence (Fig. S2I and S2J). Thus, these data suggest that SIRT7 deficiency accelerates functional attrition of hMSCs.

### SIRT7 stabilizes heterochromatin in hMSCs

SIRT7 was initially identified as a histone deacetylase capable of removing acetyl groups from acetylated H3K18 and H3K36 in cancer cells and more recently reported as a histone desuccinylase for H3K122 residues across commonly used cell lines (Barber et al., 2012; Li et al., 2016; Wang et al., 2019a). We examined these typical histone modification substrates in *SIRT7*^−/−^ hMSCs but did not find that SIRT7 depletion resulted in any detectable changes of these substrates (Fig. S2K).

**Figure 2:**
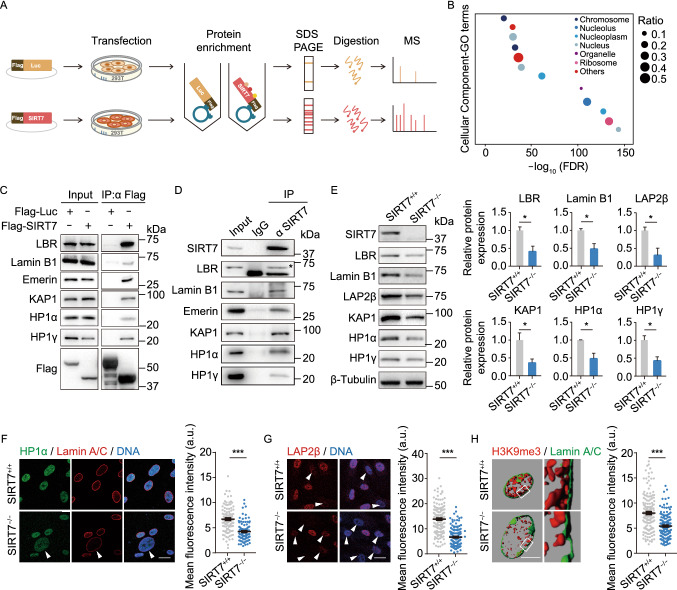
**SIRT7 interacts with nuclear lamina proteins and heterochromatin proteins**. (A) Schematic of mass spectrometry work flow for identifying SIRT7-interacting proteins. Luc was used as control. (B) Gene Ontology Cellular Component (GO-CC) enrichment analysis of candidate SIRT7-interacting proteins identified by mass spectrometry. (C) Co-immunoprecipitation analysis of LBR, Lamin B1, Emerin, KAP1, HP1α and HP1γ with exogenous Flag-tagged SIRT7 protein in HEK293T cells. (D) Co-immunoprecipitation analysis of LBR, Lamin B1, Emerin, KAP1, HP1α and HP1γ with endogenous SIRT7 protein in WT hMSCs. The band corresponding to LBR is indicated with an asterisk. (E) Left, Western blot analysis of heterochromatin-related proteins in hMSCs at MP (P6) with β-Tubulin used as loading control. Right, statistical analysis of the relative heterochromatin-related protein expression levels. Data are presented as the means ± SEM. *n* = 3. *, *P* < 0.05 (*t* test). (F) Left, immunostaining of HP1α and Lamin A/C in *SIRT7*^+/+^ and *SIRT7*^−/−^ hMSCs at MP (P6). White arrowheads indicate cells with decreased expression of HP1α. Right, mean fluorescence intensity of HP1α was measured by Image J. Scale bar, 25 μm. Data are presented as the means ± SEM. *n* = 100 cells. ***, *P* < 0.001 (*t* test). (G) Left, immunostaining of LAP2β in *SIRT7*^+/+^ and *SIRT7*^−/−^ hMSCs. White arrowheads indicate cells with decreased expression of LAP2β. Right, mean fluorescence intensity of LAP2β was measured by Image J. Scale bar, 25 μm. Data are presented as the means ± SEM. *n* = 150 cells. ***, *P* < 0.001 (*t* test). (H) Left, z-stack 3D reconstruction of H3K9me3 and Lamin A/C immunofluorescence images (shown in Fig. S3B) in *SIRT7*^+/+^ and *SIRT7*^−/−^ hMSCs at MP (P6). Scale bar, 5 μm. Right, mean fluorescence intensity of H3K9me3 in Fig. S3B was quantified with Image J. Data are presented as means ± SEM. *n* = 150 cells. ***, *P* < 0. 001 (*t* test)

To investigate a histone deacetylase or desuccinylase activity-independent role for SIRT7 in the regulation of hMSC homeostasis, we sought to identify additional interaction partners of SIRT7. To this end, we expressed Flag-tagged SIRT7 proteins in HEK293T cells and performed immunoprecipitation (IP) with an anti-Flag antibody followed by liquid chromatography-tandem mass spectrometry (LC-MS/MS) analysis (Fig. 2A and 2B). In addition to previously identified SIRT7-interacting proteins such as fibrillarin (FBL) (Iyer-Bierhoff et al., 2018), we identified a list of candidate SIRT7-interacting proteins associated with heterochromatin organization, such as nuclear lamina protein LBR and heterochromatin protein KAP1 (Fig. S3A). Furthermore, by performing co-immunoprecipitation (co-IP) assay, we identified additional nuclear lamina proteins Lamin B1 and Emerin, as well as heterochromatin proteins HP1α and HP1γ as novel SIRT7-interacting proteins (Fig. 2C and 2D). In addition, Western blot and immunofluorescence analyses revealed decreased levels of these nuclear lamina proteins (i.e., LAP2β) and heterochromatin marks (i.e., HP1α and H3K9me3) in *SIRT7*^−/−^ hMSCs relative to levels in *SIRT7*^+/+^ hMSCs (Figs. 2E–H and S3B). Consistently, we observed an increased degree of heterochromatin loss at the nuclear periphery in nuclei of *SIRT7*^−/−^ hMSCs under an electron microscope (Fig. S3C). Together, these data indicate that SIRT7 is required for maintaining heterochromatin in hMSCs.

**Figure 3:**
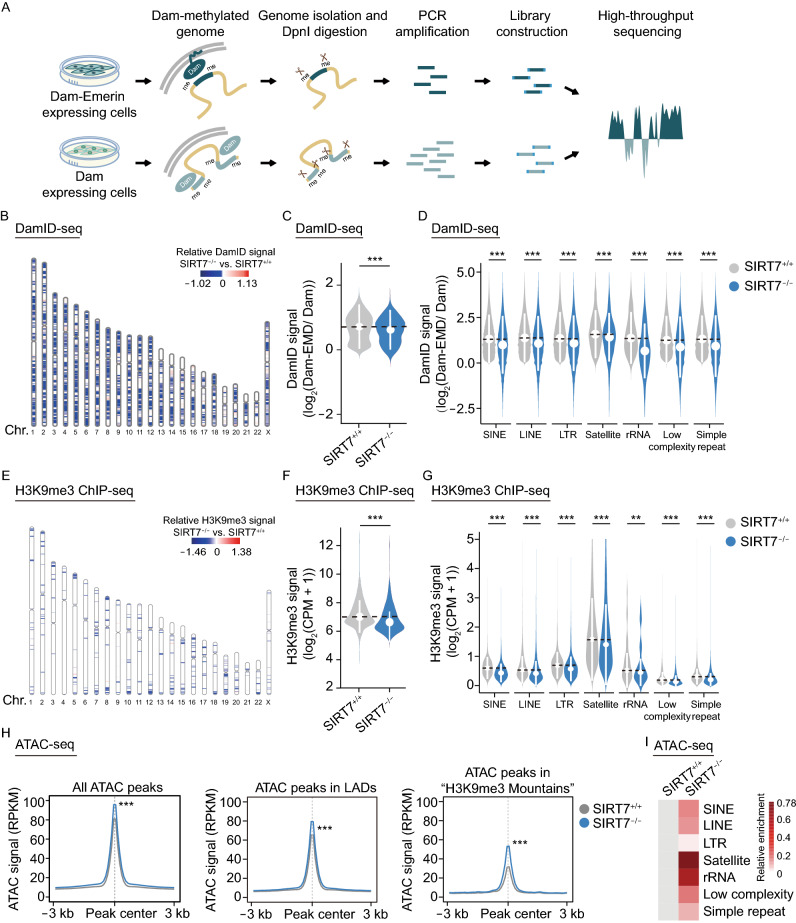
**SIRT7 is required for heterochromatin maintenance in hMSCs**. (A) Schematic diagram showing the strategy for DamID-seq library construction. When the Dam-Emerin fused protein was expressed in hMSCs, the genomic DNA region near the nuclear envelope could be methylated by DNA adenine methyltransferase (Dam) at adenines. A parallel experiment “Dam only” was used to eliminate the background Dam signal. After genome extraction, the sequence containing methylated sites could be specifically cut by DpnI and amplified by PCR. The amplified fragments were then proceeded to DNA library construction and high-throughput sequencing. (B) Chromosome ideogram showing relative DamID signals in LADs across 23 chromosomes at MP (P6). The color key from blue to red shows low to high relative DamID levels, respectively. (C) Violin plot showing the DamID signal [log_2_ (Dam-EMD/Dam)] in LADs in *SIRT7*^+/+^ and *SIRT7*^−/−^ hMSCs at MP (P6). The white circles represent the median values, and the white lines represent the values within the IQR from smallest to largest. ***, *P* < 0.001 (Two-sided Wilcoxon rank-sum test). (D) Violin plot showing the DamID signal [log_2_ (Dam-EMD/ Dam)] in LADs located in repetitive elements, including SINE, LINE, LTR, Satellite, rRNA, low complexity and simple repeat elements, in *SIRT7*^+/+^ and *SIRT7*^−/−^ hMSCs at MP (P6). The white circles represent the median values, and the white lines represent the values within the IQR from smallest to largest. ***, *P* < 0.001 (Two-sided Wilcoxon rank-sum test). (E) Chromosome ideogram showing the relative H3K9me3 signal in “H3K9me3 mountains” across 23 chromosomes at MP (P6). The color key from blue to red shows low to high relative H3K9me3 levels, respectively. (F) Violin plot showing the H3K9me3 signal in “H3K9me3 mountains” in *SIRT7*^+/+^ and *SIRT7*^−/−^ hMSCs at MP (P6). The white circles represent median values, and the white lines represent the values within the IQR from smallest to largest. ***, *P* < 0.001 (Two-sided Wilcoxon rank-sum test). (G) Violin plot showing the H3K9me3 signal in “H3K9me3 mountains” located in repetitive elements, including SINE, LINE, LTR, Satellite, rRNA, low complexity and simple repeat elements in *SIRT7*^+/+^ and *SIRT7*^−/−^ hMSCs at MP (P6). The white circles represent the median values, and the white lines represent the values within the IQR from smallest to largest. **, *P* < 0.01, ***, *P* < 0.001 (Two-sided Wilcoxon rank-sum test). (H) Metaplots showing the average ATAC signals for all ATAC peaks, ATAC peaks in LADs, and ATAC peaks in “H3K9me3 mountains” in *SIRT7*^+/+^ and *SIRT7*^−/−^ hMSCs at MP (P6). ***, *P* < 0.001 (Two-sided Wilcoxon rank-sum test). (I) Heatmap showing the relative enrichment of ATAC peaks in repetitive elements, including SINE, LINE, LTR, Satellite, rRNA, low complexity and simple repeat elements in *SIRT7*^+/+^ and *SIRT7*^−/−^ hMSCs at MP (P6). Enrichment of ATAC peaks in repetitive elements in *SIRT7*^−/−^ hMSCs were normalized to those in *SIRT7*^+/+^ hMSCs

To characterize the heterochromatin state managed by SIRT7 in greater detail, we performed DNA adenine methyltransferase identification with high-throughput sequencing (DamID-seq) that is a powerful tool to study the interactions between nuclear lamina and chromatin (Guelen et al., 2008), H3K9me3 chromatin immunoprecipitation followed by high-throughput sequencing (ChIP-seq), and chromatin accessibility assay (Assay for transposase accessible chromatin sequencing, ATAC-seq) in *SIRT7*^+/+^ and *SIRT7*^−/−^ hMSCs. DamID was performed by introducing DNA adenine methyltransferase (Dam) fused to Emerin (a nuclear lamina-associated protein), or Dam protein alone into *SIRT7*^+/+^ and *SIRT7*^−/−^ hMSCs to map the changes in LADs upon SIRT7 ablation (Fig. 3A). SIRT7 deficiency resulted in a decrease of DamID signals (Figs. 3B, 3C, and S4A–C), and the DamID signals in LAD-localized repetitive elements (LINE, SINE, LTR, Satellite and rRNA) were all diminished in *SIRT7*^−/−^ hMSCs (Fig. 3D). As expected, ChIP-seq analysis demonstrated reduced enrichment in long-range H3K9me3 occupancy (H3K9me3 mountains) in constitutive heterochromatin regions, especially at the LAD regions and the LAD located repetitive elements, indicative of a reduced association between heterochromatin and nuclear lamina (Figs. 3E–G and S4D–I). Consistent with the global heterochromatin decondensation observed in *SIRT7*^−/−^ hMSCs, ATAC-seq revealed that heterochromatin regions became more accessible when SIRT7 was depleted (Figs. 3H, 3I, and S4J–M). These data indicate a key role of SIRT7 in maintaining heterochromatin at the nuclear periphery.

### SIRT7 deficiency activates LINE1 in hMSCs

**Figure 4:**
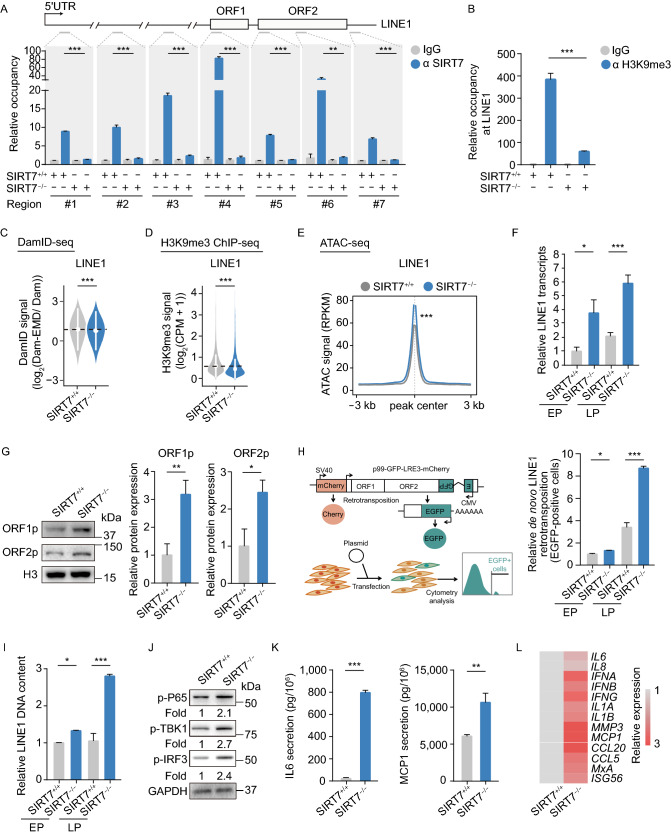
**Depletion of SIRT7 in hMSCs induces LINE1 activation and triggers innate immune responses via cGAS-STING pathway**. (A) ChIP-qPCR assessment of SIRT7 enrichment at seven LINE1 regions in *SIRT7*^+/+^ and *SIRT7*^−/−^ hMSCs at MP (P6). Data are presented as the means ± SEM. *n* = 3. **, *P* < 0.01. ***, *P* < 0.001 (*t* test). (B) ChIP-qPCR assessment of H3K9me3 enrichment of LINE1 regions in *SIRT7*^+/+^ and *SIRT7*^−/−^ hMSCs at MP (P6) using the fourth LINE1 primer. Data are presented as the means ± SEM. *n* = 4. ***, *P* < 0.001 (*t* test). (C) Violin plot showing the DamID signal [log_2_ (Dam-EMD/ Dam)] in LINE1 regions located in LADs in *SIRT7*^+/+^ and *SIRT7*^−/−^ hMSCs at MP (P6). The white circles represent the median values, and the white lines represent the values within the IQR from smallest to largest. ***, *P* < 0.001 (Two-sided Wilcoxon rank-sum test). (D) Violin plot showing the H3K9me3 signal in LINE1 regions located in “H3K9me3 mountains” in *SIRT7*^+/+^ and *SIRT7*^−/−^ hMSCs at MP (P6). The white circles represent the median values, and the white lines represent the values within the IQR from smallest to largest. ***, *P* < 0.001 (Two-sided Wilcoxon rank-sum test). (E) Metaplot showing the average ATAC signal for ATAC peaks in LINE1 regions in *SIRT7*^+/+^ and *SIRT7*^−/−^ hMSCs at MP (P6). ***, *P* < 0.001 (Two-sided Wilcoxon rank-sum test). (F) Quantitative RT-PCR analysis of LINE1 transcript levels in *SIRT7*^+/+^ and *SIRT7*^−/−^ hMSCs at EP (P3) and LP (P8). Data are presented as the means ± SEM. *n* = 4. *, *P* < 0.05. ***, *P* < 0.001 (*t* test). (G) Left, Western blot analysis of LINE1 ORF1 and ORF2 proteins in *SIRT7*^+/+^ and *SIRT7*^−/−^ hMSCs at MP (P6) with H3 used as loading control. Right, statistical analysis of relative ORF1 and ORF2 protein expression levels. Data are presented as the means ± SEM. *n* = 3. *, *P* < 0.05, **, *P* < 0.01 (*t* test). (H) Left, schematic of retrotransposition assay. The plasmid p99-GFP-LRE3-Cherry lacking a CMV promoter encodes a full-length LINE1 element tagged with an indicator cassette of mEGFP1 retrotransposition and mCherry cassette. Right, quantification of *de novo* retrotransposition events (EGFP-positive cells) in *SIRT7*^+/+^ and *SIRT7*^−/−^ hMSCs at MP (P6), normalized by live cell numbers and transfection efficiency. Data are presented as the means ± SEM. *n* = 3. *, *P* < 0.05. ***, *P* < 0.001 (*t* test). (I) RT-qPCR analysis of the relative LINE1 genomic DNA content in *SIRT7*^+/+^ and *SIRT7*^−/−^ hMSCs at EP (P3) and LP (P8). Data are presented as the means ± SEM. *n* = 4. *, *P* < 0.05. ***, *P* < 0.001 (*t* test). (J) Western blot analysis of the phosphorylation levels of P65, TBK1 and IRF3 in *SIRT7*^+/+^ and *SIRT7*^−/−^ hMSCs at MP (P6) with GAPDH used as loading control. (K) ELISA analysis of IL6 and MCP1 levels in culture medium of *SIRT7*^+/+^ and *SIRT7*^−/−^ hMSCs at MP (P6). IL6 levels were normalized to cell number. Data are presented as the means ± SEM. *n* = 5. MCP1 levels were normalized to cell number. *n* = 3. **, *P* < 0.001. ***, *P* < 0.001 (*t* test). (L) Heatmap showing quantitative RT-PCR analysis of IFN-I and SASP genes in *SIRT7*^+/+^ and *SIRT7*^−/−^ hMSCs at MP (P6). Expression levels of these indicated genes in *SIRT7*^−/−^ hMSCs were normalized to those in *SIRT7*^+/+^ hMSCs

Constitutive heterochromatin is normally required for the inactivation of retrotransposons such as LINE1 (De Cecco et al., 2013; Gorbunova et al., 2014; Van Meter et al., 2014). Using ChIP-qPCR assay, we detected that SIRT7 bound specific LINE1 promoter regions in *SIRT7*^+/+^ but not in *SIRT7*^−/−^ hMSCs (Fig. 4A). As we observed an increase in accessibility at a variety of genomic repetitive elements, ChIP-qPCR indicated a decrease in H3K9me3 enrichment at LINE1 promoter when SIRT7 was absent (Fig. 4B). The combined results from DamID-seq, H3K9me3 ChIP-seq, and ATAC-seq analyses supported the notion that deficiency of SIRT7 in hMSCs led to de-repression and increased chromatin accessibility of LINE1 (Fig. 4C–E). Real-time quantitative PCR (RT-qPCR) indeed demonstrated increased RNA levels of LINE1 in *SIRT7*^−/−^ hMSCs (Fig. 4F). Consistent with the elevated transcription activity of LINE1 in *SIRT7*^−/−^ hMSCs, we found that LINE1 coding proteins ORF1 and ORF2 were upregulated both in *SIRT7*^−/−^ hMSCs and physiologically senescent primary hMSCs (Figs. 4G and S5A). To examine whether the retrotransposition activity of LINE1 is increased in the absence of SIRT7, we conducted retrotransposition analysis in *SIRT7*^+/+^ and *SIRT7*^−/−^ hMSCs with an engineered LINE1 reporter system (Thomas et al., 2017). As shown in Fig. 4H, a higher retrotransposition frequency of LINE1 was observed in *SIRT7*^−/−^ hMSCs relative to *SIRT7*^+/+^ hMSCs. We also detected an elevated genomic DNA content of LINE1 in *SIRT7*^−/−^ hMSCs with RT-qPCR (Fig. 4I). These results indicate that SIRT7 deficiency enables increased transcription of LINE1 and subsequent LINE1-dependent retrotransposition events.

### Activation of cGAS-STING pathway and innate immune responses in SIRT7-deficient hMSCs

Given reports on LINE1 activation functioning as a fuse for the DNA sensing cGAS-STING signaling pathway (Volkman and Stetson, 2014; De Cecco et al., 2019; Geng et al., 2019; Simon et al., 2019), we next interrogated its potential association with SIRT7 deficiency and activation of LINE1. Activation of the cGAS-STING pathway was evident in SIRT7-deficient hMSCs, as detected by increased levels of phosphorylated forms of NF-κB P65, TANK-binding kinase 1 (TBK1) and IFN regulatory factor 3 (IRF3) (Fig. 4J). Correspondingly, elevated gene expression related to type-I interferon (IFN-I) and SASP responses, as downstream effectors of cGAS-STING signaling was observed in *SIRT7*^−/−^ hMSCs (Figs. 4K, 4L, and S2G), indicating that SIRT7 deficiency contributes to the appearance of a range of senescence-related phenotypes, at least in part by activation of LINE1 events and the cGAS-STING pathway.

### SIRT7 can be targeted to rejuvenate aged hMSCs

**Figure 5:**
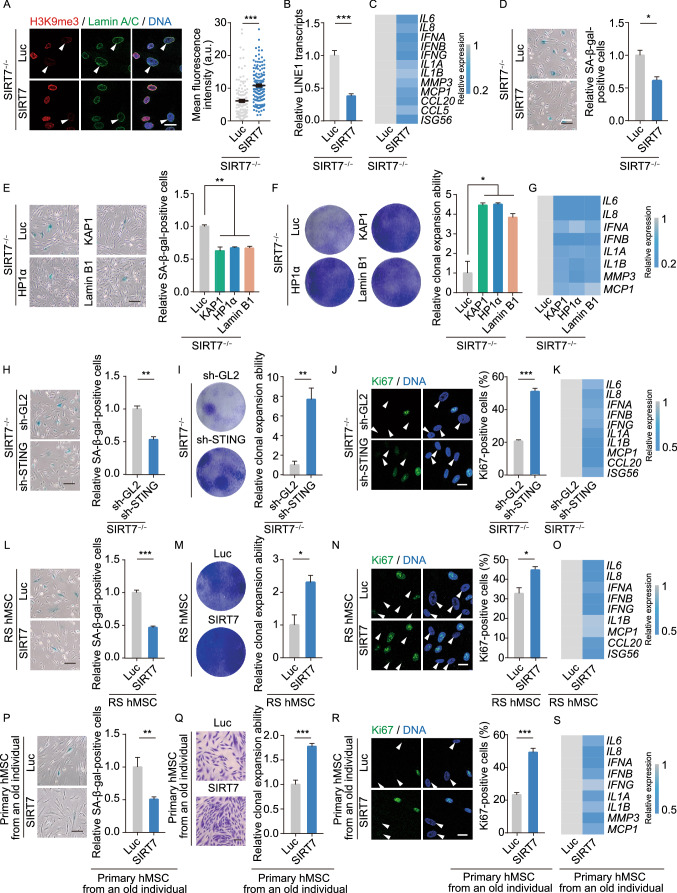
**SIRT7-heterochromatin-cGAS-STING axis protects hMSCs against senescence**. (A) Left, immunostaining of H3K9me3 and Lamin A/C in *SIRT7*^−/−^ hMSCs transduced with lentiviruses expressing Luc or SIRT7. White arrowheads indicate cells with decreased expression of H3K9me3. Scale bar, 25 μm. Right, mean fluorescence intensity of H3K9m3 was measured by Image J. Data are presented as the means ± SEM. *n* = 150 cells. ***, *P* < 0.001 (*t* test). (B) Quantitative RT-PCR analysis of LINE1 transcript levels in *SIRT7*^*−/−*^ hMSCs transduced with lentiviruses expressing Luc or SIRT7. Data are presented as the means ± SEM. *n* = 4. ***, *P* < 0.001 (*t* test). (C) Heatmap showing quantitative RT-PCR analysis of IFN-I and SASP genes in *SIRT7*^−/−^ hMSCs transduced with lentiviruses expressing Luc or SIRT7. Expression levels of these indicated genes in SIRT7 lentivirus-transduced hMSCs were normalized to those in Luc lentivirus-transduced hMSCs. (D) SA-β-gal staining of *SIRT7*^−/−^ hMSCs transduced with lentiviruses expressing Luc or SIRT7. Scale bar, 125 μm. Data are presented as means ± SEM. *n* = 3. *, *P* < 0.05 (*t* test). (E) SA-β-gal staining of *SIRT7*^−/−^ hMSCs transduced with lentiviruses expressing KAP1, HP1α or Lamin B1. Scale bar, 125 μm. Data are presented as the means ± SEM. *n* = 3. **, *P* < 0.01 (*t* test). (F) Clonal expansion analysis of *SIRT7*^−/−^ hMSCs transduced with lentiviruses expressing KAP1, HP1α or Lamin B1. Data are presented as the means ± SEM. *n* = 3. *, *P* < 0.05 (*t* test). (G) Heatmap showing quantitative RT-PCR analysis of IFN-I and SASP genes in physiologically senescent hMSCs from old donor transduced with lentiviruses expressing KAP1, HP1α or Lamin B1. Expression levels of these indicated genes in KAP1, HP1α or Lamin B1-transduced hMSCs were normalized to those in Luc-transduced hMSCs. (H) SA-β-gal staining of *SIRT7*^−/−^ hMSCs transduced with lentiviruses expressing sh-GL2 or sh-STING. Scale bar, 125 μm. Data are presented as the means ± SEM. *n* = 3. **, *P* < 0.01 (*t* test). (I) Clonal expansion analysis of *SIRT7*^−/−^ hMSCs transduced with lentiviruses expressing sh-GL2 or sh-STING. Data are presented as the means ± SEM. *n* = 3. **, *P* < 0.01 (*t* test). (J) Immunostaining of Ki67 in *SIRT7*^−/−^ hMSCs transduced with lentiviruses expressing sh-GL2 or sh-STING. White arrowheads indicate cells with decreased expression of Ki67. Scale bar, 25 μm. Data are presented as the means ± SEM. *n* = 3. ***, *P* < 0.001 (*t* test). (K) Heatmap showing quantitative RT-PCR analysis of IFN-I and SASP genes in *SIRT7*^−/−^ hMSCs transduced with lentiviruses expressing sh-GL2 or sh-STING. Expression levels of these indicated genes in sh-STING lentivirus-transduced hMSCs were normalized to those in sh-GL2 lentivirus-transduced hMSCs. (L) SA-β-gal staining of RS hMSCs transduced with lentiviruses expressing Luc or SIRT7. Scale bar, 125 μm. Data are presented as the means ± SEM. *n* = 3. ***, *P* < 0.001 (*t* test). (M) Clonal expansion analysis of RS hMSCs transduced with lentiviruses expressing Luc or SIRT7. Data are presented as the means ± SEM. *n* = 3. *, *P* < 0.05 (*t* test). (N) Immunostaining of Ki67 in RS hMSCs transduced with lentiviruses expressing Luc or SIRT7. White arrowheads indicate cells with decreased expression of Ki67. Scale bar, 25 μm. Data are presented as the means ± SEM. *n* = 3. *, *P* < 0.05 (*t* test). (O) Heatmap showing quantitative RT-PCR analysis of IFN-I and SASP genes in RS hMSCs transduced with lentiviruses expressing Luc or SIRT7. Expression levels of these indicated genes in SIRT7 lentivirus-transduced hMSCs were normalized to those in Luc lentivirus-transduced hMSCs. (P) SA-β-gal staining of old primary hMSCs from an 80-year-old individual (Old #2) transduced with lentiviruses expressing Luc or SIRT7. Scale bar, 125 μm. Data are presented as the means ± SEM. *n* = 3. **, *P* < 0.01 (*t* test). (Q) Clonal expansion analysis of old primary hMSCs from an 80-year-old individual (Old #2) transduced with lentiviruses expressing Luc or SIRT7. Data are presented as the means ± SEM. *n* = 5. ***, *P* < 0.001 (*t* test). (R) Immunostaining of Ki67 in old primary hMSCs from an 80-year-old individual (Old #2) transduced with lentiviruses expressing Luc or SIRT7. White arrowheads indicate cells with decreased expression of Ki67. Scale bar, 25 μm. Data are presented as the means ± SEM. *n* = 3. ***, *P* < 0.001 (*t* test). (S) Heatmap showing quantitative RT-PCR analysis of IFN-I and SASP genes in old primary hMSCs from an 80-year-old individual (Old #2) transduced with lentiviruses expressing Luc or SIRT7. Expression levels of these indicated genes in SIRT7 lentivirus-transduced hMSCs were normalized to those in Luc lentivirus-transduced hMSCs

To investigate how the epistasis between SIRT7, heterochromatin loss, LINE1 activation, and induction of the cGAS-STING pathway coordinate acquisition of hMSC senescence, we performed the following experiments. First, we restored the expression of SIRT7 in *SIRT7*^−/−^ hMSCs via lentiviral vector-mediated gene transfer. As expected, re-introduction of SIRT7 restored H3K9me3 levels, reduced LINE1 transcription, and attenuated IFN-I and SASP responses, and rescued phenotypes of accelerated hMSC senescence. We observed decreased percentages of SA-β-gal-positive cells, increased clonal expansion ability, and a increase in Ki67-positive proliferating cells (Figs. 5A–D, S5B, and S5C). Second, considering that nuclear lamina proteins (i.e., Lamin B1) and heterochromatin proteins (i.e., KAP1 and HP1α) as newly identified SIRT7 interaction partners were downregulated in *SIRT7*^−/−^ hMSCs, we examined how ectopic expression of Lamin B1 and heterochromatin proteins KAP1 and HP1α affected SIRT7-deficient cells. Similar to re-introduction of SIRT7, restoring Lamin B1, KAP1 or HP1α expression in *SIRT7*^−/−^ hMSCs alleviated aging defects, including decreased percentages of SA-β-gal-positive cells, increased proliferative potential, and attenuated expression of IFN-I and SASP genes (Figs. 5E–G, S5D, and S5E). Third, we asked whether blocking the cGAS-STING pathway would rescue the senescence phenotypes caused by SIRT7 deficiency. shRNA knockdown of STING in *SIRT7*^−/−^ hMSCs led to alleviation of the senescence phenotypes, resembling overexpression of SIRT7 or individual heterochromatin components (Figs. 5H–K and S5F). Collectively, these data converge on a SIRT7-heterochromatin-cGAS-STING axis as an important geroprotective mechanism in hMSCs.

Finally, we explored whether targeting SIRT7 might rejuvenate replicatively and physiologically senescent primary hMSCs isolated from an 80-year-old individual. Indeed, we found that restoring SIRT7 similarly alleviated the senescence phenotypes we had observed in SIRT7-null hMSCs, including decreased percentages of SA-β-gal-positive cells, improved cellular expansion ability, increased Ki67-positive cells, and attenuated expression of IFN-I and SASP genes (Fig. 5L–S).

### 3TC is a geroprotective compound for hMSCs

**Figure 6:**
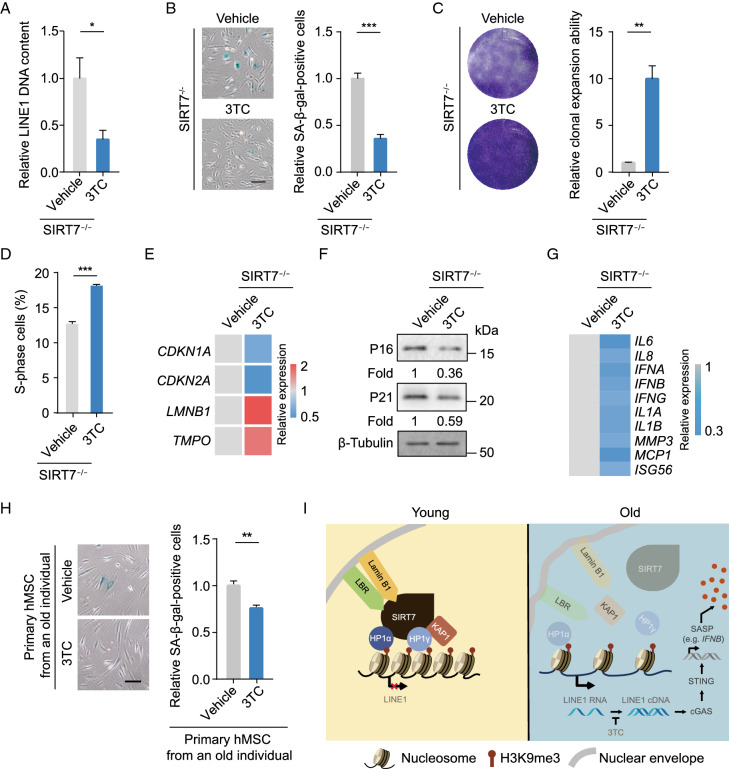
**3TC attenuates SIRT7-deficient hMSCs senescence by antagonizing LINE1’s effects**. (A) RT-qPCR analysis of the relative LINE1 genomic DNA content in *SIRT7*^−/−^ hMSCs treated with vehicle or 3TC. Data are presented as the means ± SEM. *n* = 4. *, *P* < 0.05 (*t* test). (B) SA-β-gal staining of *SIRT7*^−/−^ hMSCs treated with vehicle and 3TC. Scale bar, 125 μm. Data are presented as means ± SEM. *n* = 3. ***, *P* < 0.001 (*t* test). (C) Clonal expansion analysis of *SIRT7*^−/−^ hMSCs treated with vehicle and 3TC. Data are presented as means ± SEM. *n* = 3. **, *P* < 0.01 (*t* test). (D) Bar plot showing the percentages of cells in S-phase of cell cycle in *SIRT7*^−/−^ hMSCs treated with vehicle or 3TC. Data are presented as the means ± SEM. *n* = 3. ***, *P* < 0.001 (*t* test). (E) Heatmap showing quantitative RT-PCR analysis of aging-related markers in *SIRT7*^−/−^ hMSCs treated with vehicle or 3TC. Expression levels of these indicated genes in 3TC-treated hMSCs were normalized to those in vehicle-treated hMSCs. (F) Western blot analysis of P16 and P21 proteins in *SIRT7*^−/−^ hMSCs treated with vehicle or 3TC with β-Tubulin used as loading control. (G) Heatmap showing quantitative RT-PCR analysis of IFN-I and SASP genes in *SIRT7*^−/−^ hMSCs treated with vehicle or 3TC. Expression levels of these indicated genes in 3TC-treated hMSCs were normalized to those in vehicle-treated hMSCs. (H) SA-β-gal staining of old primary hMSCs from an 80-year-old individual (Old #2) treated with vehicle or 3TC. Scale bar, 125 μm. Data are presented as means ± SEM. *n* = 3. **, *P* < 0.01 (*t* test). (I) A model illustrating SIRT7-mediated heterochromatin stabilization in hMSCs. In young hMSCs, SIRT7 functions as a stabilizer for nuclear lamina proteins Lamin B1, LBR, and heterochromatin proteins KAP1, HP1α and HP1γ, which together form a complex at LADs to ensure chromatin spatial organization and silence LINE1. In old hMSCs, SIRT7 downregulation leads to the detachment of the complex from LADs and promotes the transcription and accumulation of LINE1 DNA, which in turn triggers activation of the cGAS-STING pathway and an immune response. 3TC is used to inhibit LINE1 reverse transcription and thus rescue senescence phenotypes

Reverse-transcriptase inhibitors (RTis) are nucleoside analogs that can inhibit reverse transcriptase, among which the anti-HIV1 drug Lamivudine (3TC) has been shown to inhibit reverse transcription of LINE1 (Jones et al., 2008; Dai et al., 2011). We, therefore, wondered whether 3TC-mediated reduction of the LINE1 life cycle might rescue senescence phenotypes in *SIRT7*^−/−^ hMSCs. The treatment of *SIRT7*^−/−^ hMSCs with 10 μmol/L 3TC led to the downregulation of the LINE1 genomic DNA content (Fig. 6A), and rescued cellular senescence phenotypes, as detected by reduced percentages of SA-β-gal-positive cells, increased proliferative potential, increased percentages of S-phase cells, upregulated transcript levels of *LMNB1* and *TMPO*, reduced expression of aging markers *CDKN1A* (P21) and *CDKN2A* (P16), and reduced aging-associated immune activation (as detected by reduced IFN-I and SASP responses) (Fig. 6B–G). More importantly, 3TC treatment alleviated senescence phenotypes in physiologically senescent hMSCs isolated from aged individuals (Fig. 6H). Our findings reveal a critical role of the cGAS-STING pathway in mediating geroprotection downstream of SIRT7, but also point to the possibility of developing 3TC as a therapy for alleviating stem cell senescence and treating aging-related disorders (Fig. 6I).

## Discussion

In this study, we closed the knowledge gap between nucleolar sirtuin SIRT7 function and hMSC senescence, revealing that SIRT7 is required for the association between the nuclear lamina and heterochromatin regions and epigenetic silencing of LINE1. Below are the highlights of our study: (1) We generated *SIRT7*^−/−^ hESCs and hMSCs through CRISPR/Cas9 technology. (2) The expression of SIRT7 was downregulated during aging, which in turn functioned as a driver for hMSC senescence. (3) SIRT7 maintained heterochromatin architecture via complexing with nuclear lamina proteins and heterochromatin components. (4) Depletion of SIRT7 induced LINE1 activation and triggered senescence phenotypes via activating cGAS-STING pathway in hMSCs. (5) 3TC attenuated hMSC senescence as a LINE1’s inhibitor.

SIRT7 is reported to govern a wide array of biological processes, frequently through its enzymatic activity. For instance, SIRT7 is recruited to DNA damage sites and catalyzes both deacetylation of H3K18ac and desuccinylation of H3K122succ to protect genome integrity (Li et al., 2016; Vazquez et al., 2016). Besides, SIRT7 can act as a deglutarylase of H4K91glu, the downregulation of which is tightly associated with chromatin remodeling and DNA repair (Bao et al., 2019). Intrigued by such diverse roles of SIRT7 in different species and cellular contexts, we set out to explore novel functions of SIRT7 in human stem cells. We thereby turned to CRISPR/Cas9 gene-editing to create SIRT7-null hESCs and further differentiated them into hMSCs. In line with previous work showing that hESCs are tolerant of a variety of cellular defects caused by genetic and epigenetic aberrations (Zhang et al., 2013), we found that SIRT7 was dispensable for hESC pluripotency. In contrast, SIRT7-deficient hMSCs were vulnerable to replicative stress and showed accelerated senescence and various aging defects. Nevertheless, it is interesting to note that depletion of SIRT7 in hMSCs did not result in changes in its typical histone substrates, suggesting the existence of cell type-specific or compensatory mechanisms for endogenous SIRT7. SIRT7 complexed with nuclear lamina proteins and heterochromatin proteins, which was mechanistically required for stable heterochromatin architecture at the nuclear periphery.

To our knowledge, we provide the first evidence that SIRT7 regulated hMSC senescence by modulating a heterochromatin-LINE1-cGAS-STING axis. Emerging evidence indicates that condensed heterochromatin is needed to safeguard genomic integrity, at least in part by silencing LINE1. For example, KAP1 facilitates the formation of repressive heterochromatin and initiates epigenetic silencing of LINE1 in early development stage (Castro-Diaz et al., 2014; Percharde et al., 2018). Moreover, SIRT6 was found to ribosylate KAP1 for silencing LINE1 in mouse embryonic fibroblasts (MEFs) (Van Meter et al., 2014). In our study, SIRT7 exerted a similar repressive effect on LINE1 yet through a different mechanism by which it facilitated the association of LINE1-contained heterochromatin to the nuclear lamina. Likewise, Serrano and co-workers recently observed the interplay between SIRT7 and LINE1 in MEFs (Vazquez et al., 2019). However, no involvement of SIRT7 in geroprotection and innate immune regulation was reported in their study. Our work imposes increased emphasis on the link between downstream mechanisms involving the activation of the cGAS-STING pathway and human stem cell aging. The cGAS-STING pathway is a sensor for accumulation of cytoplasmic DNA and activates the innate immune response. Our study, along with two very recent papers (De Cecco et al., 2019; Simon et al., 2019), suggests that increased LINE1 activity drives cGAS-STING pathway activation and induces cellular senescence. SIRT6, another nuclear-localized sirtuin family member, is not only capable of protecting hMSCs from oxidative injuries by coactivating transcription factor NRF2 (Pan et al., 2016) but also promotes healthy lifespan of mice through LINE1-cGAS-STING axis (Simon et al., 2019). SIRT6 and SIRT7 are both nuclear-localized and able to repress the LINE1-cGAS-STING axis to protect against cellular senescence. Consistent with the results that 3TC improves the health and cellular pathologies in Sirt6-deficient mice (Simon et al., 2019), we found that 3TC could ameliorate the premature aging phenotypes of SIRT7-deficient hMSCs. Our study extends the knowledge of sirtuins in regulating heterochromatin and downstream cGAS-STING pathway, which not only points to a role of SIRT7 loss in driving hMSC aging but also provides a plausible explanation for the emergence of other hMSC senescence-associated phenotypes including loss of heterochromatin, activation of LINE1, and induction of cGAS-STING pathway and related SASP.

Perhaps the most intriguing finding is that the downstream molecular programs of SIRT7 may be targeted with a small-molecule inhibitor to achieve the reversal of cellular aging. We show in this study that either lentiviral overexpression of SIRT7 or application of the small-molecule chemical 3TC rejuvenated aged hMSCs. Since hMSC senescence is functionally linked to various human aging syndromes (Zhang et al., 2015; Kubben et al., 2016; Deng et al., 2019; Fu et al., 2019; Ren et al., 2019), the approaches generated here (i.e., compound intervention and gene therapy) may be applicable to identify other compounds or molecular targets for the development of future therapies for age-related disease.

## Materials and methods

### Cell culture

WT-hESCs (Line H9, from WiCell Research) and *SIRT7*^−/−^ hESCs were maintained on mitomycin C-inactivated MEFs in hESC culture medium (Cheng et al., 2019): 80% DMEM/F12 (Gibco), 20% Knockout Serum Replacement (Gibco), 2 mmol/L GlutaMAX (Gibco), 0.1 mmol/L non-essential amino acids (NEAA, Gibco), 55 μmol/L β-mercaptoethanol (Invitrogen), and 10 ng/mL bFGF (Joint Protein Central); hESCs were also cultured on Matrigel (BD Biosciences) with mTeSR medium (STEMCELL Technologies, Vancouver). All hMSCs were cultured in MSC culture medium: 90% α-MEM with GlutaMAX (Gibco), 10% fetal bovine serum (FBS, Lot A77E01F, Gemcell), 1% penicillin/streptomycin (Gibco) and 1 ng/mL bFGF (Joint Protein Central). 10 μmol/L Lamivudine (3TC) (L1295-50mg, Sigma-Aldrich) was used to treat with hMSCs.

### Knockout of *SIRT7* gene in WT hESCs

CRISPR/Cas9-mediated gene editing (Suzuki et al., 2016) was performed as previously described (Wang et al., 2020) with a variety of modifications. In brief, *SIRT7* gRNA (CAGCGTCTATCCCAGACTAC) targeting exon 4 of *SIRT7* was cloned into gRNA-mCherry vector (*SIRT7*-gRNA-mCherry). The pCAG-1BPNLS-Cas9-1BPNLS-2AGFP plasmid was purchased from Addgene (#87109). After electroporation, cells were seeded on Matrigel-coated plates and treated with ROCK inhibitor (Tocris) in mTeSR. After 48 h of expansion, dual-positive cells were collected by FACS (BD FACS Aria II) and plated on MEF feeder cells in hESC medium. Emerging clones were manually picked into 24-well plates and then genomic DNAs of the clones were extracted for PCR and sequencing. The corrected hESC clones were homozygous for the addition of base A, resulting in TAA premature termination at both alleles and thus leading to complete knockout of *SIRT7*. hESC clones were then expanded on Matrigel-coated plates and gene knockout was confirmed by Western blot analysis. Primer sequences are shown in Table S1.

### Generation of hMSCs

Embryoid bodies were left to differentiate in hMSC differentiation medium (Yan et al., 2019) supplemented with MEMα Gibco, 10% FBS (Lot A77E01F, Gemcell), 10 ng/mL bFGF (Joint Protein Central), and 5 ng/mL TGFβ (Humanzyme) until fibroblast-like cells appeared. hESC-derived hMSCs were then FACS-purified with different antibodies corresponding to hMSC-specific markers (CD73, CD90, and CD105). Surface antigens CD44, CD166, CD29, HLA-ABC and CD13 were used as hMSC positive markers, and CD164, CD14, CD19 and PDPN as hMSC negative markers. The tri-lineage differentiation potential of hMSC lines was evaluated using von Kossa (osteogenesis), Alcian blue (chondrogenesis), and Oil red O kit (IHC World) (adipogenesis) (Wang et al., 2018).

### Lentiviral shRNA and CRISPR/Cas9-mediated gene knockdown

STING-specific shRNAs cloned into pLVTHM plasmids (Addgene, #12247) were co-transfected with psPAX2 (Addgene, #12260) and pMD2.G (Addgene, #12259) plasmids into HEK293T cells for lentivirus production and viruses were then collected with ultracentrifugation at 19,400 ×*g* for 2.15 h. SIRT7-specific sgRNAs were cloned into lenti-CRISPRv2 (Addgene, #52961) with an hSpCas9 expression cassette for Lentiviral CRISPR/Cas9-mediated gene editing. Lentivirus-mediated depletion of SIRT7 was performed in WT hMSCs. For the enrichment of lentivirus-transduced cells, cells were treated with 0.5 μg/mL puromycin (Gibco) at 72 h after infection. Primer sequences are shown in Table S2.

### Isolation and culture of primary hMSCs

Primary hMSCs were isolated from different individuals at the No. 306 Hospital of PLA, under the approval by the ethics committee (Zhang et al., 2015; Deng et al., 2019; Fu et al., 2019; Ren et al., 2019; Yan et al., 2019). Dental pulp tissues were processed into small pieces in TrypLE™ Express Enzyme (1×) plus Dispase IV and further digested at 37 °C for 30 min. The mixture was filtered by a cell strainer (70 μm), and then centrifuged at 500 ×*g* for 10 min (RT). The pellets consist of supernatant were cultivated on Gelatin-coated plates with MSC culture medium overnight. The culture medium for hMSCs was changed every other day. The sample information is shown in Table S2.

### Western blot analysis

Cells were lysed in 2× SDS buffer (Sigma-Aldrich) for analysis of non-phosphorylated proteins, and in RIPA buffer for analysis of phosphorylated proteins. To measure the protein concentration, we used the BCA Kit from Thermo Fisher Scientific. Approximately 20 μg protein per sample was subjected to SDS-PAGE electrophoresis and then transferred to PVDF membranes (Millipore). After a blocking step with 5% milk (BBI Life Sciences), membranes were incubated with different primary antibodies and corresponding HRP-conjugated secondary antibodies. The quantification of image was used by Image Lab software (ChemiDoc XRS+ system, Bio-Rad).

### Immunofluorescence microscopy

Cells seeded on coverslips (Thermo Fisher Scientific) were washed twice in PBS and fixed in 4% paraformaldehyde (PFA) for 30 min. 0.4% Triton X-100 (Sigma) was diluted by PBS, in which cells were permeabilized for 30 min. Then, cells were blocked in 10% donkey serum in PBS (Jackson ImmunoResearch) for 1 h (RT). After incubation with primary antibodies in blocking buffer at 4 °C overnight, cells were incubated with secondary antibodies at room temperature for 1 h and labeled with Hoechst33342 (Thermo Fisher Scientific) to visualize nuclei. Images were taken with a Leica SP5 confocal microscope.

### Transmission electron microscope (TEM)

WT and SIRT7-deficient hMSCs at middle passage (P6) were harvested by TrypLE™ (Thermo Fisher Scientific) and centrifuged at 500 ×*g* for 5 min (RT). The collected pellets were fixed with 4% PFA in PBS, pH 7.4, on ice overnight. Using graded series of ethanol, cells were dehydrated and then infiltrated with Lowicryl resin HM20. Images were collected with a Spirit transmission electron microscope (FEI Company) operating at 100 kV.

### Plasmid construction

To generate plasmids overexpressing SIRT7, KAP1, HP1α, Lamin B1 or luciferase (Luc, used as control), cDNAs were cloned into the pLE4 vector (a gift from Tomoaki Hishida) (Deng et al., 2019).

### SA-β-gal staining

SA-β-gal staining was performed as previously described (Ren et al., 2019). Briefly, cultured cells were fixed for 5 min (RT) in the stationary liquid mixed by 0.2% glutaraldehyde and 2% formaldehyde. Fixed cells were stained with SA-β-gal staining solution at 37 °C overnight, percentages of SA-β-gal-positive cells were then calculated.

### Clonal expansion assay

Clonal expansion assays were performed as previously described (Fu et al., 2019). Briefly, 2,000 MSCs were seeded in a Gelatin-coated 12-well plate. Harvested cells were fixed in 4% paraformaldehyde (PFA) for 30 min, and stained with 0.2% crystal violet for 1 h (RT). Relative cell density was quantified with Image J.

### Measurement of ROS level

For ROS measurements, living cells were incubated with ROS indicators (1 μmol/L CM-H2DCFDA, C6827, Molecular Probes). All measurements were achieved with an LSRFortessa cell analyzer (BD), and the data were analyzed with FlowJo software (TreeStar, Ashland, OR).

### Co-immunoprecipitation (co-IP)

HEK293T cells were transfected with plasmids expressing Flag-Luc (control) and Flag-SIRT7. Cells were then lysed in the lysis buffer (120 mmol/L NaCl, 0.3% CHAPS, 1 mmol/L EDTA, 40 mmol/L HEPES, pH 7.5, and complete protease inhibitor cocktail (Roche)). *SIRT7*^+/+^ hMSCs were lysed in CHAPS lysis buffer for endogenous co-IP. HEK293T cells or hMSCs were lysed at 4 °C for 2 h and then centrifuged at 12,000 ×*g* at 4 °C for 30 min. For endogenous co-IP, lysates (1 mg protein) were pre-cleared with 20 μL of Protein A/G-PLUS Agarose beads (Santa Cruz) for 2 h, and the supernatants were then collected by centrifugation at 3,000 rpm at 4 °C for 3 min. Next, supernatants were mixed with indicated antibodies and the beads were rotated at 4 °C overnight. The immunocomplexes were washed three times with CHAPS buffer and then eluted by boiling in 2× SDS-loading buffer for 10 min.

### LC-MS/MS analysis

Protein eluates from IP were separated by 10% SDS-PAGE gel. The gel was stained with Coomassie brilliant blue and then washed with water for several times to destain. After determining molecular weight, bands were excised from the gel for aimed proteins. Samples then underwent dehydration (100% acetonitrile), reduction (10 mM DTT in 25 mmol/L NH_4_HCO_3_ for 45 min at 56 °C) and alkylation (40 mmol/L iodoacetamide in 25 mmol/L NH_4_HCO_3_ for 45 min at room temperature in the dark). After sequence-grade modified trypsin (40 ng for each band) in 25 mmol/L NH_4_HCO_3_, gel bands were dried and digested. Finally, the enzymatic reaction was stopped with formic acid. The resultant solution was identified using nanoLC-Q EXACTIVE (Thermo Scientific) equipped with data-dependent mode that allows MS data acquirement at a high resolution 70,000 (m/z 200) across the mass range of 300–1,600 m/z. Using Sequest HT and search engine, all raw files were processed from Q Exactive and analyzed with Proteome Discovery version 1.4 for protein identification and Percolator for FDR (false discovery rate). Our data were analyzed against a Uniprot human protein database (updated on 06-2013). Percolator used FDR analysis. We defined FDR <1% for protein identification. The confidence of peptides was set as high for peptides filter. Gene Ontology enrichment analysis was conducted by ToppGene (Chen et al., 2009).

### Antibodies

The antibodies used for Western blot analysis are as follows: anti-SIRT7 (#5360S, 1:1,000), anti-HP1α (#2616S, 1:1,000) anti-HP1γ (#2619, 1:3,000), anti-p-TBK1 (#5483S, 1:1,000), anti-p-IRF3 (#4947S, 1:1,000), anti-p-P65 (#3033S, 1:1,000), anti-P21 (#2947S, 1:1,000) and anti-H3K36ac (07-354, 1:1,000) from *Cell Signaling Technology*, anti-KAP1 (Ab22553, 1:2,000), anti-LBR (Ab32535, 1:1,000), anti-Lamin B1 (Ab16048, 1:1,000), anti-LINE1-ORF2p (Ab106004, 1:500) and anti-H3K18ac (Ab1191, 1:2,000) from *Abcam*, anti-β-Tubulin (sc-5274, 1:3,000) and anti-H3 (sc-10809, 1:2,000) from *Santa Cruz Biotechnology*, anti-LAP2 (611000, 1:500) and anti-P16 (550834, 1:500) from *BD Biosciences*, anti-Flag (#F1804, 1:3,000) and anti-H3K36ac (07-354, 1:1,000) from *Sigma-Aldrich*, and anti-H3K122succ (PTM-413 1:3,000) from *PTM BioLabs*.

The antibodies used for immunostaining are as follows: anti-Ki67 (ZM0166, 1:500) from *ZSGB-BIO*, anti-phospho-Histone H2AX (Ser139) (05636, 1:400), anti-LINE1-ORF1p (MABC1152, 1:1,000) from *Millipore*, anti-53BP1 (A300-273A, 1:600) from *Bethyl Laboratories*, anti-Lamin A/C (sc-376248, 1:500), anti-OCT3/4 (sc-5279, 1:200) and anti-SOX2 (sc-17320, 1:100) from *Santa Cruz Biotechnology*, anti-H3K9me3 (Ab8898, 1:500) and anti-NANOG (Ab21624, 1:200) from *Abcam*, and anti-FABP4 (AF3150, 1:100) from *R&D Systems*.

The antibodies used for flow cytometry are as follows: anti-CD73 (550741, 1:200), anti-CD90 (555595, 1:200), anti-CD14 (555398, 1:200), anti-CD44 (550989, 1:200), anti-CD43, and anti-CD19 (555415, 1:200) from *BD Biosciences*, anti-CD105 (17-1057, 1:200) and anti-PDPN (17-9381-41, 1:200) from *eBioscience*, anti-CD29 (303004, 1:200), anti-CD166 (343903, 1:200) and anti-CD164 (324805, 1:200) from *Biolegend*.

### DNA and RNA analyses

Total RNA at early (P3) and late passages (P8) was extracted in TRIzol™ (Thermo Fisher Scientific), reverse transcribed to cDNA using GoScript™ Reverse Transcription System for RT-qPCR (Promega) and subjected to genomic DNA removal using a DNA-free kit (Thermo Fisher Scientific). Genomic DNA was then purified with a DNA extraction kit (TIANGEN), and PCR was carried out with PrimeSTAR polymerase. RT-qPCR was performed using the qPCR Mix (TOYOBO) in a CFX384 Real-Time system (Bio-Rad). Primer sequences are shown in Table S1.

### RNA-seq library construction and sequencing

Using the NEBNext® Poly (A) mRNA Magnetic Isolation Module, mRNA was isolated for RNA-seq. We constructed sequencing libraries using the NEBNext® Ultra™ RNA Library Prep Kit for Illumina following the manufacturer’s protocol and then sequenced libraries on Illumina HiSeq X-Ten platforms with paired-end 150-bp sequencing. Quality control and sequencing were done by Novogene Bioinformatics Technology.

### RNA-seq data processing

Raw reads for RNA-seq libraries in WT and SIRT7-deficient cells were trimmed by the Trim Galore software (version 0.4.5) (https://github.com/FelixKrueger/TrimGalore). We then aligned cleaned reads to the UCSC human hg19 genome using hisat2 (version 2.0.4) (Kim et al., 2015). Then, the expression level (read count) for each gene was calculated counted with HTSeq (version 0.11.0), keeping only high-quality reads (mapping quality more than 20) (Anders et al., 2015). Fragments Per Kilobase per Million (FPKM) was calculated using stringTie (version 1.2.3) (Pertea et al., 2015). Gene set enrichment analysis (GSEA) was conducted by GSEA software (version 2.2.4) (Subramanian et al., 2005).

### DamID-seq

DamID-seq was performed as described (Vogel et al., 2007) with modifications. The plasmids pLgw V5-EcoDam and pLgw EcoDam-V5-EMD were gifts from Prof. Bas van Steensel, NKI. WT MSCs and *SIRT7*^−/−^ MSCs at P6 were transduced with Dam or Dam-EMD lentivirus. Cells were harvested after 72 h and the genomic DNA was isolated using DNeasy Blood & Tissue Kit (Qiagen). After DpnI digestion, adaptor ligation, DpnII digestion, PCR amplification and purification, the amplified DNA was sonicated and digested with AlwI (NEB) to remove the adaptors. The DNA libraries were constructed using a NEBNext® Ultra™ DNA Library Prep Kit and were then pooled and subjected to 150 bp paired-ends sequencing on Illumina NovaSeq sequencers.

### DamID-seq data processing

For the processing of DamID-seq data, raw reads were trimmed by the Trim Galore software (version 0.4.5) (https://github.com/FelixKrueger/TrimGalore). Cleaned reads were aligned to UCSC human hg19 genome using Bowtie 2 (version 2.2.9) (Langmead and Salzberg, 2012). Duplicated reads were filtered out by MarkDuplicates.jar program in Picard tools (https://broadinstitute.github.io/picard/). To minimize the bias of sequencing depth, replicates for each sample were merged. Then, 120 million high-quality reads for Dam or Dam-EMD data set in WT or *SIRT7*^−/−^ hMSCs were randomly selected and retained for downstream analysis. To visualize the DamID signal, we calculated the log_2_ transformed Reads Per Kilobase per Million mapped reads (RPKM) values of Dam-EMD and Dam signal [log_2_(Dam-EMD/Dam)] in WT or *SIRT7*^−/−^ hMSCs for each 10-bp bin using bamCompare program deepTools (version 2.5.4-2-5ee467f) software.

To identify LAD regions in WT or *SIRT7*^−/−^ hMSCs, we calculated log_2_ transformed RPKM values of Dam-EMD and Dam signal [log_2_ (Dam-EMD/Dam)] in WT or *SIRT7*^−/−^ hMSCs for each 2-kb bin. We then identified LAD regions with the R package HMMt (https://github.com/dinovski/asDamID/blob/master/scripts/hmmt_functions.R).

### ChIP-seq and ChIP-qPCR

ChIP-seq and ChIP-qPCR were performed following a previously reported protocol with minor modifications (Deng et al., 2019). Briefly, WT and *SIRT7*^−/−^ hMSCs at middle passage (P6) were cross-linked in 1% formaldehyde at room temperature with continuous rotation for 10 min. Next, cross-linked cells were quenched with 125 mmol/L Glycine for 5 min and then lysed on ice for 10 min, followed by chromatin shearing to a target peak size of 100–500 bp using the Covaris S220 focused-ultrasonicator (Covaris). The supernatant retained after centrifugation was then incubated with 2.4 μg H3K9me3 antibody (Ab8898, *Abcam*) or SIRT7 antibody (#5360S, *Cell Signaling Technology*) pre-conjugated with Dynabeads Protein A (10002D, Thermo Fisher Scientific) overnight at 4 °C. Normal rabbit IgG (#2729S, *Cell Signaling Technology*) was used as a negative control. After elution and reverse crosslinking at 68 °C, fragmented DNA was purified with phenol-chloroform-isoamyl alcohol extraction and ethanol precipitation and subjected to sequencing or RT-qPCR analysis. Libraries were prepared using KAPA Hyper Prep Kits (KK8504, KAPA Biosystems) and Next Multiplex Oligos for Illumina (Index Primers Set 1) (E7335L, New England Biolabs), and then sequenced on HiSeq X-Ten platforms according to the manufacturer’s instruction. Primer sequences are shown in Table S1.

### ChIP-seq data processing

Raw reads were trimmed using the Trim Galore software (version 0.4.5) (https://github.com/FelixKrueger/TrimGalore) and clean reads aligned to the UCSC human hg19 genome using Bowtie 2 (version 2.2.9) (Langmead and Salzberg, 2012). To minimize the bias of sequencing depth, replicates for each sample were merged. Then, 60 million high-quality reads were randomly selected and retained for downstream analysis. We then called H3K9me3 peaks by SICER (version 1.1) with the parameter “-w 200 -g 3” (Zang et al., 2009). H3K9me3 peaks with FDR (False discovery rate) of 1% or better were retained.

To identify “H3K9me3 mountains”, H3K9me3 signal (CPM, Count Per Million) for each H3K9me3 peak in WT or *SIRT7*^−/−^ hMSCs was calculated. We then ranked H3K9me3 peaks by increasing H3K9me3 signal in WT or *SIRT7*^−/−^ hMSCs as shown in Fig. S4E. These plots showed inflection points where the H3K9me3 signal began increasing rapidly. H3K9me3 peaks above the inflection point were then named as “H3K9me3 mountains”.

### ATAC-seq library preparation and sequencing

ATAC-seq libraries of hMSCs were prepared as previously described (Buenrostro et al., 2013; Wu et al., 2016; Wang et al., 2019b) with some modifications. In brief, 50,000 cells of WT or *SIRT7*^−/−^ hMSCs at middle passage (P6) were washed twice in cold PBS and lysed in 50 μL ice-cold lysis buffer (10 mmol/L Tris-HCl (pH 7.4), 10 mmol/L NaCl, 3 mmol/L MgCl_2_ and 0.5% NP-40) for 10 min. Lysates were briefly spun at 500 ×*g* for 5 min to obtain the nuclei. The nuclei were then incubated with the Tn5 transposome and tagmentation buffer at 37 °C for 30 min (TD501, Vazyme Biotech). After tagmentation, fragmented DNA was purified with 100 μL (2×) AMPure XP beads (A63882, Beckman Coulter) and amplified for 13 cycles using the following PCR conditions: 72 °C for 3 min; 98 °C for 30 s; and thermocycling at 98 °C for 15 s, 60 °C for 30 s and 72 °C for 30 s; following by 72 °C 5 min. PCR reaction enzyme and index primers were purchased from Vazyme Biotech (TD202). After PCR amplification, libraries were purified and selected by using 0.5× /1.3× AMPure XP beads. Finally, libraries were sequenced on HiSeq X-Ten platforms according to the manufacturer’s instruction.

### ATAC-seq data processing

Raw reads were cleaned with the Trim Galore software (version 0.4.5) and trimmed reads aligned to the UCSC human hg19 genome using Bowtie 2 (version 2.2.9) with the parameter “ -X 2,000 -N 1 -L 25 –no-mixed –no-discordant -t ” (Langmead and Salzberg, 2012). Reads of PCR duplicates were removed with the MarkDuplicates.jar program in Picard tools. Replicates for each sample were merged for downstream analysis. For ATAC peak calling, MACS2 (version 2.1.1) was used with the parameter “–nomodel –shift 0 –extsize 250 –call-summits” (Zhang et al., 2008).

### Whole genome sequencing and copy number variation analysis

Genomic DNA from 1 × 10^6^ WT or *SIRT7*^−/−^ hMSCs at middle passage (P6) as per duplicate was isolated using DNeasy Blood & Tissue Kit (Qiagen). Quality control and sequencing were performed following standard protocols from Novogene Bioinformatics Technology Co. Ltd. Genome-wide copy number variation (CNV) analysis was conducted as previously described (Deng et al., 2019). Raw reads were trimmed by the TrimGalore software (version 0.4.5) and clean reads aligned to the UCSC hg19 human genome using bowtie2 software (version 2.2.9). R package HMMcopy (version 1.25.0) was implemented to calculate CNVs in each 0.5 Mb bin size (Ha et al., 2012).

### Assessment of the reproducibility of sequencing data

To evaluate the reproducibility of H3K9me3 ChIP-seq and ATAC-seq data sets, we first calculated RPKM level for each 2-kb bin genome widely. We then calculated the Euclidean distance by R (version 3.5.1) to evaluate reproducibility. Lower value of Euclidean distance means highly correlated. To evaluate the reproducibility of DamID-seq, principle component analysis (PCA) was calculated by R (version 3.5.1). Pearson correlation coefficient (R) between replicates was calculated based on the expression level (regularized-logarithm normalized read count) to evaluate the reproducibility of RNA-seq data.

### Retrotransposition assay

WT and *SIRT7*^−/−^ hMSCs were transfected with 2 μg p99-GFP-LRE3-Cherry (a gift from Alysson R. Muotri) and EGFP-positive cells were sorted by FACS at three days post-transfection. We calculated the adjusted retrotransposition rate by number of EGFP-positive cells normalized by the transfection efficiency, which was determined by the mCherry reporter, and by transfected cell numbers.

### ELISA

Protein levels of IL6 and MCP1 were measured by sandwich enzyme-linked immunoassay (ELISA). Supernatants from WT and *SIRT7*^−/−^ hMSCs at middle passage (P6) were collected and centrifuged at 500 ×*g* for 5 min to remove debris. ELISA kits from systems were used: IL6 (BioLegend) and MCP1 (R&D Systems). Plates were scanned at 450 nm using Synergy H1 (BioTek) and finally data were normalized by cell numbers.

### Comet assay

Comet assay was performed as described previously (Olive and Banath, 2006) with a minor modification. The images were acquired by CKX41 microscope and then quantified using CASP software.

### Animal experiments

hMSC implantation assays were performed as previously described (Fu et al., 2019). One million *SIRT7*^+/+^ or *SIRT7*^−/−^ hMSCs expressing luciferase were injected into the TA muscle of male nude mice aged 6-8 weeks. *In vivo* luciferase activity was measured by an IVIS Spectrum imaging system (XENOGEN, Caliper).

### Statistical analysis

All data in our study were presented as the means ± SEM. We used GraphPad Prism 7 Software to conduct two tailed Student’s *t*-test. *P* value < 0.05 was considered statistically significant.

## Electronic supplementary material

Below is the link to the electronic supplementary material.

Supplementary material 1 (PDF 3969 kb)

Supplementary material 2 (XLSX 13 kb)

Supplementary material 3 (XLSX 11 kb)

Supplementary material 4 (XLSX 160 kb)

Supplementary material 5 (XLSX 62 kb)

## Abbreviations

3TC: Lamivudine
ATAC-seq: Assay for transposase accessible chromatin sequencing
cGAS-STING: The cyclic GMP-AMP synthase-stimulator of interferon genes
CNV: Genome-wide copy number variation
co-IP: Co-immunoprecipitation
CRISPR/Cas9: Clustered regularly interspaced short palindromic repeat/CRISPR associated gene 9
DamID-seq: DNA adenine methyltransferase identification with high-throughput sequencing
DSBs: DNA double-strand breaks
DDR: DNA damage response
H3K9me3: Histone H3 Lys9 trimethylation
HDACs: Histone deacetylases
hESC: Human embryonic stem cell
HGPS: Hutchinson-Gilford progeria syndrome
hMSC: Mesenchymal stem cell
LC-MS/MS: Liquid chromatography-tandem mass spectrometry
LADs: Lamina-associated domains
LINE1: Long interspersed element 1
Luc: Luciferase
MEF: Mouse embryonic fibroblast
NAD: Nicotinamide adenine dinucleotide
PDPN: Podoplanin
RTis: Reverse-transcriptase inhibitors
ROS: Reactive oxygen species
RS: Replicatively senescent
SA-β-gal: Senescence-associated-β-galactosidase
SASP: Senescence-associated secretory phenotype
TA: Tibialis anterior
TE: Transposable element
WT: Wild-type
