# Correction to: SIRT7 antagonizes human stem cell aging as a heterochromatin stabilizer

**DOI:** 10.1093/procel/pwad037

**Published:** 2023-07-15

**This is a correction to:** Shijia Bi and others, SIRT7 antagonizes human stem cell aging as a heterochromatin stabilizer, *Protein & Cell*, Volume 11, Issue 7, July 2020, Pages 483–504, https://doi.org/10.1007/s13238-020-00728-4

The authors wish to introduce the following corrections to their article.

Supplementary Fig. S1F showed the proportion of Ki67 on *SIRT7*^+/+^ and *SIRT7*^−/−^ hESCs, where the top representative image was accidentally copied and pasted as the bottom representative image. A new supplementary Fig. S1 is provided below.


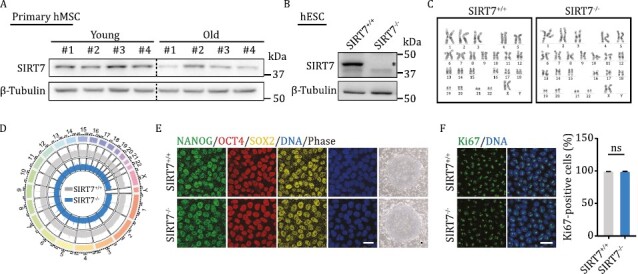


**Figure S1. Characterization of SIRT7-deficient hESCs.** (A) Western blot analysis of SIRT7 protein in young and old primary hMSCs with β-Tubulin used as loading control. (B) Western blot analysis of SIRT7 protein in *SIRT7*^+/+^ and *SIRT7*^−/−^ hESCs with β-Tubulin used as loading control. The band corresponding to SIRT7 was indicated with an asterisk. (C) Karyotyping analysis of *SIRT7*^+/+^ and *SIRT7*^−/−^ hESCs. (D) Genome-wide analysis of copy number variations (CNVs) in *SIRT7*^+/+^ and *SIRT7*^−/−^ hESCs. (E) Immunostaining of OCT4, SOX2, and NANOG and bright-field in *SIRT7*^+/+^ and *SIRT7*^−/−^ hESCs. Scale bar, 25 μm. (F) Immunostaining of Ki67 in *SIRT7*^+/+^ and *SIRT7*^−/−^ hESCs. Scale bar, 25 μm. Data are presented as the means ± SEM. *n* = 3. ns, not significant (*t* test).

In supplementary table (Table S1) for primers, the primers for LINE1 were in wrong order. A new supplementary Table S1 is provided and the primer information is shown below.

Table S1. The primers used for PCR, RT-qPCR and ChIP-qPCR.

**Table:** 

Name	Sequences
LINE1-1-Forward	AAGATGGCCGAATAGGAACAG
LINE1-1-Reverse	TTTGACTCGGAAAGGGAACTC
LINE1-2-Forward	ACGAGACTATATCCCACACCT
LINE1-2-Reverse	GCAGAGGTTACTGCTGTCTT
LINE1-3-Forward	TGTCTGACAGCTTTGAAGAGAG
LINE1-3-Reverse	TGGTCTTTGATGATGGTGATGTA
LINE1-4-Forward	CGATGCGATCAACTGGAAG
LINE1-4-Reverse	GGCCTGCCTTGCTAGATT
LINE1-5-Forward	CAGAGACACACATAGGCTCAAA
LINE1-5-Reverse	AATCTGGGTGCTCCTGTATTG
LINE1-6-Forward	ACTCATCTGACAAAGGGCTAAT
LINE1-6-Reverse	CCTATTTCTCCGCATCCTCTC
LINE1-7-Forward	AATGAGATCACATGGACACAGGAAG
LINE1-7-Reverse	TGTATACATGTGCCATGCTGGTGC

These emendations are outlined and provided only in this correction notice to preserve the version of record.

