# Correction to: SIRT7 antagonizes human stem cell aging as a heterochromatin stabilizer

**DOI:** 10.1093/procel/pwaf031

**Published:** 2025-05-20

This is a correction to: Shijia Bi, Zunpeng Liu, Zeming Wu, Zehua Wang, Xiaoqian Liu, Si Wang, Jie Ren, Yan Yao, Weiqi Zhang, Moshi Song, Guang-Hui Liu, Jing Qu, SIRT7 antagonizes human stem cell aging as a heterochromatin stabilizer, *Protein & Cell*, Volume 11, Issue 7, July 2020, Pages 483–504, https://doi.org/10.1007/s13238-020-00728-4

A recent internal review by the authors identified an inadvertent oversight in the KAP1 panel of Fig. S5D, where an image was not accurately presented. While this does not impact the study’s conclusions or overall discussion, the corrected supplementary figure is presented here:


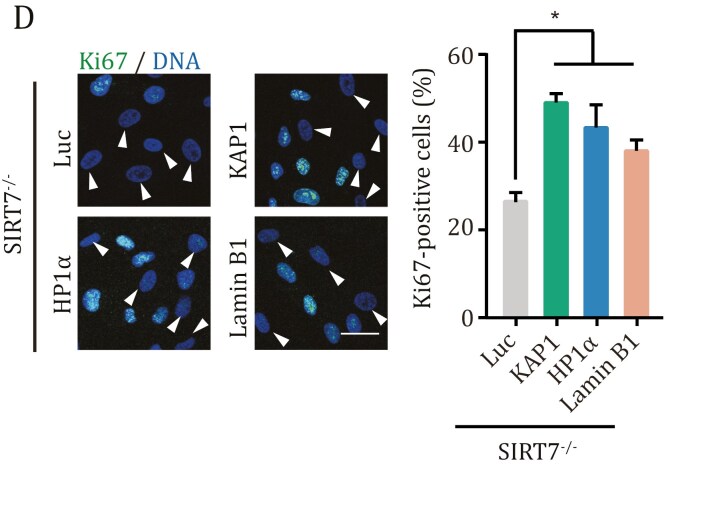


These details have been corrected only in this correction notice to preserve the published version of record.

