# Supplementary material for: SIRT7 antagonizes human stem cell aging as a heterochromatin stabilizer
Source: Protein Cell. 2020 Jun 6;11(7):483–504. doi: 10.1007/s13238-020-00728-4 (PMC7305295; doi:10.1007/s13238-020-00728-4)
Supplement: Supplementary file 1 — Supplementary material 1 (PDF 3969 kb) [file 13238_2020_728_MOESM1_ESM.pdf]

Figure S1. Characterization of SIRT7-deficient hESCs

(A) Western blot analysis of SIRT7 protein in young and old primary hMSCs with β-Tubulin used as loading control.
(B) Western blot analysis of SIRT7 protein in *SIRT7*$^{+/+}$ and *SIRT7*$^{-/-}$ hESCs with β-Tubulin used as loading control. The band corresponding to SIRT7 was indicated with an asterisk.
(C) Karyotyping analysis of *SIRT7*$^{+/+}$ and *SIRT7*$^{-/-}$ hESCs.
(D) Genome-wide analysis of copy number variations (CNVs) in *SIRT7*$^{+/+}$ and *SIRT7*$^{-/-}$ hESCs.
(E) Immunostaining of OCT4, SOX2, and NANOG and bright-field in *SIRT7*$^{+/+}$ and *SIRT7*$^{-/-}$ hESCs. Scale bar, 25 μm.
(F) Immunostaining of Ki67 in *SIRT7*$^{+/+}$ and *SIRT7*$^{-/-}$ hESCs. Scale bar, 25 μm. Data are presented as the means ± SEM. n = 3. n.s., not significant (t test).

Figure S2. Characterization of SIRT7-deficient hMSCs

(A) Table of surface antigen analysis of *SIRT7*$^{+/+}$ and *SIRT7*$^{-/-}$ hMSCs. Data are presented as means ± SEM. n = 3.
(B) Characterization of adipogenesis, osteogenesis and chondrogenesis in *SIRT7*$^{+/+}$ and *SIRT7*$^{-/-}$ hMSCs. Scale bar, 125 μm. FABP4 staining and Oil Red O staining were used to evaluate adipogenesis. Von kossa staining was used to evaluate osteogenesis. Toluidine blue staining was used to evaluate chondrogenesis.
(C) Immunostaining of γH2AX and 53BP1 in *SIRT7*$^{+/+}$ and *SIRT7*$^{-/-}$ hMSCs at MP (P6). Scale bar, 10 μm. Data are presented as the means ± SEM. n = 150 cells. ***, $p < 0.001$ (t test).
(D) Comet assay in *SIRT7*$^{+/+}$ and *SIRT7*$^{-/-}$ hMSCs at MP (P6). Data are presented as the means ± SEM. n = 50 cells. ***, $p < 0.001$ (t test).
(E) Heatmap showing the Euclidean distance analysis to evaluate the reproducibility of RNA-seq in *SIRT7*$^{+/+}$ and *SIRT7*$^{-/-}$ hMSCs at EP (P3). The color key of the Euclidean distance from red to blue indicates strong to weak correlation, respectively.
(F) Gene set enrichment analysis (GSEA) showing enrichment of the term “aging” in *SIRT7*$^{+/+}$ and *SIRT7*$^{-/-}$ hMSCs at EP (P3).
(G) GSEA showing enrichment of the terms “IFN-I” and “SASP” in *SIRT7*$^{+/+}$ and *SIRT7*$^{-/-}$ hMSCs at EP (P3).
(H) Western blot analysis of SIRT7 protein in *SIRT7*$^{-/-}$ hMSCs transduced with lentiviruses expressing sg-NTC, sg-SIRT7 #1, or sg-SIRT7 #2 with β-Tubulin used as loading control.
(I) SA-β-gal staining of *SIRT7*$^{-/-}$ hMSCs transduced with lentiviruses expressing sg-NTC, sg-SIRT7 #1, or sg-SIRT7 #2. Scale bar, 125 μm. Data are presented as the means ± SEM. n = 3. **, $p < 0.01$ (t test).
(J) Clonal expansion analysis of *SIRT7*$^{-/-}$ hMSCs transduced with lentiviruses expressing sg-NTC, sg-SIRT7 #1, or sg-SIRT7 #2. Data are presented as the means ± SEM. n = 3. *, $p < 0.05$ (t test)
(K) Western blot analysis of H3K18ac, H3K36ac and H3K122succ levels in *SIRT7*$^{+/+}$ and *SIRT7*$^{-/-}$ hMSCs with H3 used as loading control.

Figure S3. SIRT7 is a stabilizer of heterochromatin complexes

(A) Table of interacting unique peptides proteins of SIRT7 by MS. Luc was used as a control.
(B) Immunostaining of H3K9me3 and Lamin A/C in *SIRT7*$^{+/+}$ and *SIRT7*$^{-/-}$ hMSCs at MP (P6). White arrowheads indicate cells with decreased expression of H3K9me3. Scale bar, 25 μm.
(C) Electron microscope (EM) analysis of the heterochromatin architecture at peripheral nucleus in *SIRT7*$^{+/+}$ and *SIRT7*$^{-/-}$ hMSCs at MP (P6). The percentages of cells with dense heterochromatin at the nuclear periphery are shown in the right. Scale bar, 2 μm.

Figure S4. Epigenomic analyses of SIRT7-deficient hMSCs

(A) Principal components analysis of the reproducibility of DamID-seq in *SIRT7*$^{+/+}$ and *SIRT7*$^{-/-}$ hMSCs at MP (P6).

(B) Histogram showing genomic coverage of LADs regions in *SIRT7*$^{+/+}$ and *SIRT7*$^{-/-}$ hMSCs at MP (P6).
(C) Heatmap of DamID signals [$\log_2$ (Dam-EMD/ Dam)] ranging from 0.15 Mb upstream to 0.15 Mb downstream of LADs regions in *SIRT7*$^{+/+}$ and *SIRT7*$^{-/-}$ hMSCs at MP (P6).
(D) Heatmap showing the Euclidean distance analysis to evaluate the reproducibility of H3K9me3 ChIP-seq in *SIRT7*$^{+/+}$ and *SIRT7*$^{-/-}$ hMSCs at MP (P6). The color key of the Euclidean distance from red to blue indicates strong to weak correlation, respectively.
(E) Distribution of H3K9me3 ChIP-seq signals at H3K9me3 peaks reveals two classes of H3K9me3 peaks in *SIRT7*$^{+/+}$ and *SIRT7*$^{-/-}$ hMSCs at MP (P6). H3K9me3 peaks are plotted in an increasing order based on H3K9me3 signal. “H3K9me3 Mountains” were defined as the population of H3K9me3 peaks above the inflection point of the curve.
(F) Bar plot showing genomic coverage of “H3K9me3 mountains” in *SIRT7*$^{+/+}$ and *SIRT7*$^{-/-}$ hMSCs at MP (P6).
(G) Heatmap of H3K9me3 ChIP-seq signals (RPKM) ranging from 3 kb upstream to 3 kb downstream of “H3K9me3 mountains” regions in *SIRT7*$^{+/+}$ and *SIRT7*$^{-/-}$ hMSCs at MP (P6).
(H) Bar plot showing the percentage of “H3K9me3 mountains” located at LADs regions in *SIRT7*$^{+/+}$ and *SIRT7*$^{-/-}$ hMSCs.
(I) Violin plot of DamID signals [$\log_2$ (Dam-EMD/ Dam)] in “H3K9me3 mountains” in *SIRT7*$^{+/+}$ and *SIRT7*$^{-/-}$ hMSCs at MP (P6). The white circles represent the median values, and the white lines represent the values within the IQR from smallest to largest. ***, $p < 0.001$ (two-sided Wilcoxon rank-sum test).
(J) Heatmap showing the Euclidean distance analysis to evaluate the reproducibility of ATAC-seq in *SIRT7*$^{+/+}$ and *SIRT7*$^{-/-}$ hMSCs at MP (P6). The color key of the Euclidean distance from red to blue indicates strong to weak correlation, respectively.
(K) Bar plot showing genomic coverage of ATAC peaks in *SIRT7*$^{+/+}$ and *SIRT7*$^{-/-}$ hMSCs at MP (P6).
(L) Bar plot showing the percentage of genomic elements that overlapping of ATAC peaks identified in *SIRT7*$^{+/+}$ and *SIRT7*$^{-/-}$ hMSCs at MP (P6).
(M) Heatmap showing ATAC signals in all ATAC peaks, LADs located ATAC peaks, and “H3K9me3 mountains” located ATAC peaks in *SIRT7*$^{+/+}$ and *SIRT7*$^{-/-}$ hMSCs at MP (P6).

Figure S5. Characterization of rescue strategy in SIRT7-deficient hMSCs
(A) Left, western blot analysis of LINE1 ORF1 and ORF2 proteins in young and old primary hMSCs with β-Tubulin used as loading control. Right, statistical analysis of the relative LINE1 ORF1 and ORF2 protein expression levels. Data are presented as the means ± SEM. n = 4 samples. *, $p < 0.05$ (t test).
(B) Immunostaining of Ki67 in *SIRT7*$^{-/-}$ hMSCs transduced with lentiviruses expressing Luc or SIRT7. White arrowheads indicate cells with decreased expression of Ki67. Scale bar, 25 μm. Data are presented as the means ± SEM. n = 3. **, $p < 0.01$ (t test).
(C) Clonal expansion analysis of *SIRT7*$^{-/-}$ hMSCs transduced with lentiviruses expressing Luc or SIRT7. Data are presented as the means ± SEM. n = 3. *, $p < 0.05$ (t test).
(D) Immunostaining of Ki67 in *SIRT7*$^{-/-}$ hMSCs transduced with lentiviruses expressing KAP1, HP1α or Lamin B1. White arrowheads indicate cells with decreased expression of Ki67. Scale bar, 25 μm. Data are presented as the means ± SEM. n = 3. *, $p < 0.05$ (t test).
(E) Bar plot showing the percentages of cells in S-phase cells in *SIRT7*$^{-/-}$ hMSCs transduced with lentiviruses expressing KAP1, HP1α or Lamin B1. Data are presented as the means ± SEM. n = 3. *, $p < 0.05$ (t test).
(F) Western blot analysis of STING protein in *SIRT7*$^{-/-}$ hMSCs transduced with lentiviruses expressing sh-GL2 or sh-STING with β-Tubulin used as loading control.

SUPPLEMENTARY TABLE LEGENDS：

Table S1. The primers used for PCR, RT-qPCR and ChIP-qPCR.
Table S2. DNA sequences of sgRNAs and shRNAs used for gene knockout or knockdown,

along with the sample information of primary hMSCs.
Table S3. Candidates identified as SIRT7-interacting proteins by mass spectrometry, related to Fig. 2.
Table S4. DEGs identified by RNA-seq analysis of *SIRT7*$^{+/+}$ and *SIRT7*$^{-/-}$ hMSCs, related to Fig. S2.

Figure S1

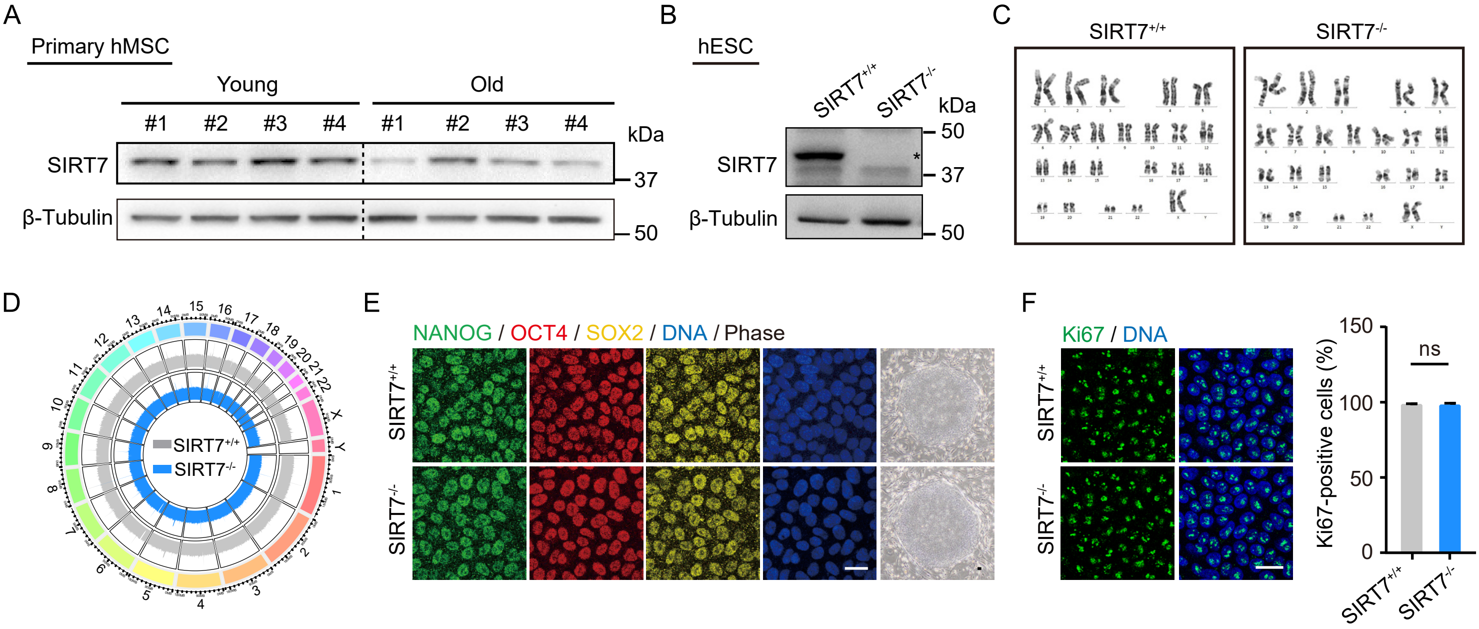

A
Primary hMSC
Young
Old
#1
#2
#3
#4
#1
#2
#3
#4
kDa
SIRT7
37
β-Tubulin
50
B
hESC
SIRT7+/+
SIRT7-/-
kDa
50
SIRT7
*
37
β-Tubulin
50
C
SIRT7+/+
SIRT7-/-
D
SIRT7+/+
SIRT7-/-
E
NANOG / OCT4 / SOX2 / DNA / Phase
SIRT7+/+
SIRT7-/-
F
Ki67 / DNA
SIRT7+/+
SIRT7-/-
Ki67-positive cells (%)
150
100
50
0
ns
SIRT7+/+
SIRT7-/-

Figure S2

A

Surface antigen	Positive cells (%)	
	SIRT7$^{+/+}$	SIRT7$^{-/-}$
CD105	98.30 ± 0.12	97.20 ± 0.21
CD90	97.10 ± 0.26	97.40 ± 0.24
CD73	98.50 ± 0.08	97.45 ± 0.17
CD44	98.19 ± 0.12	99.44 ± 0.02
CD166	99.62 ± 0.02	99.12 ± 0.53
CD29	99.26 ± 0.11	99.45 ± 0.17
CD164	0.91 ± 0.02	2.69 ± 0.01
CD14	0.10 ± 0.08	0.17 ± 0.04
CD19	0.02 ± 0.01	0.10 ± 0.07
PDPN	0.12 ± 0.07	0.08 ± 0.04

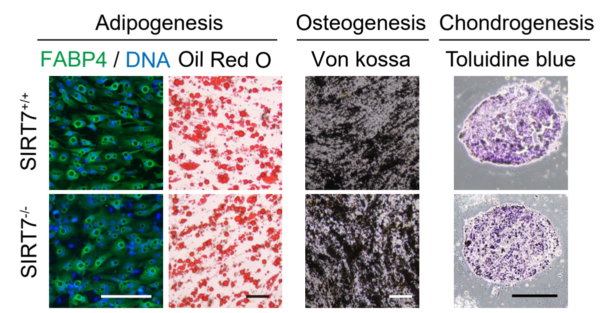

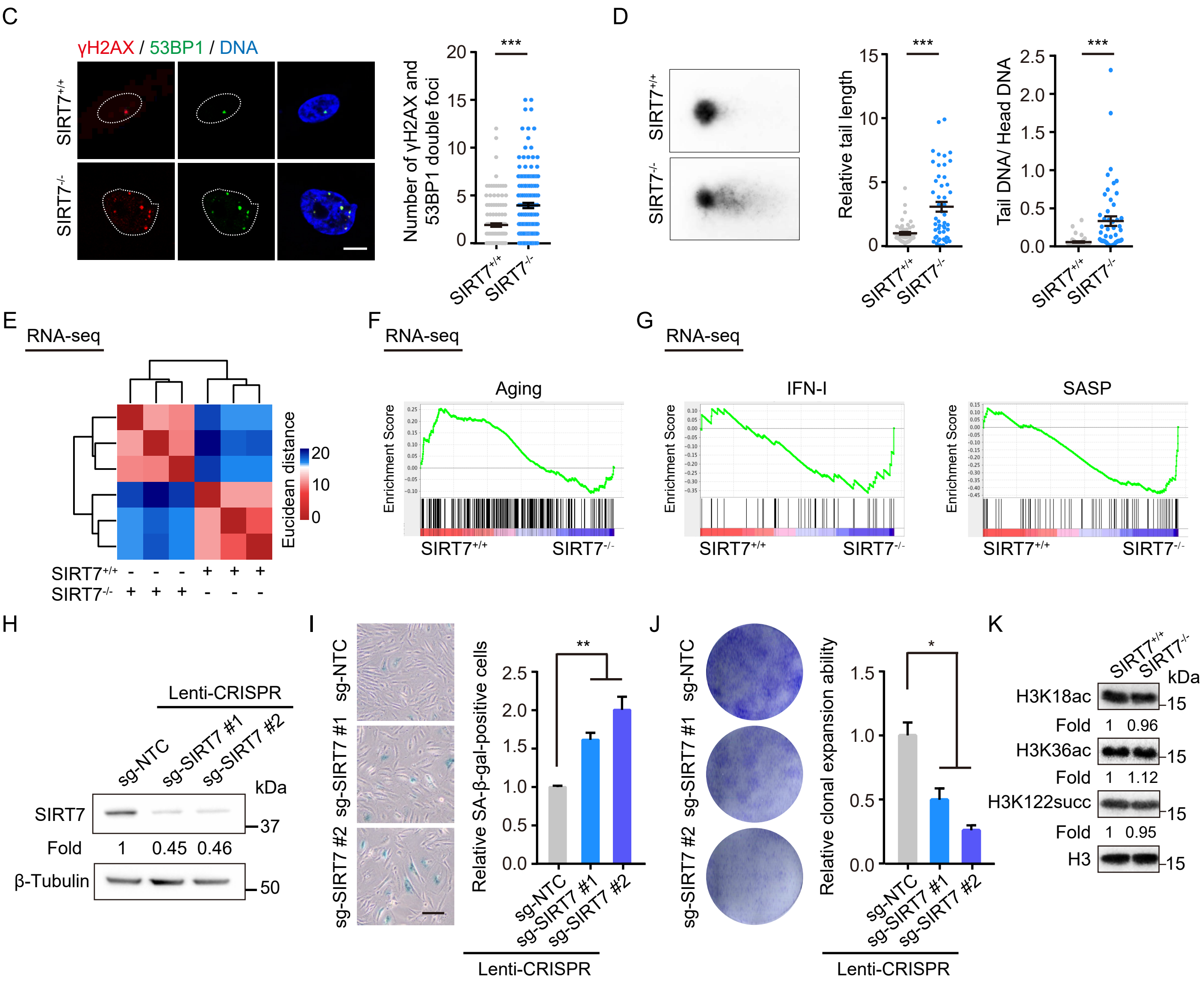

Figure S3

A

Name	Function	Coverage (%)	Unique peptides	Peptide sequence
TRIM28 (KAP1)	Heterochromatin maintenance	18.08	14	IVAERPGTNSTGPAPmAPPR<br>LTEDKADVQSIIGLQR<br>LDLDLTADSQPPVFK<br>MAILQIMK<br>VFPGSTTEDYNLIVIER<br>FQWDLNAWTK<br>ADVQSIIGLQR<br>LIYFQLHR<br>SGEGEVSGLMR<br>LSPPYSSPQEFAQDVGR<br>DIVENYFMR<br>MIVDPVEPHGEMK<br>LLASLVK<br>SGEGEVSGLmR
LBR	Chromatin interaction	7.04	1	LTPLILKPFGNSISR
FBL	Nucleolar component	36.45	9	RVSISEGDDKIEYR<br>ANcIDSTASAEAVFASEVKK<br>DHAVVVGVYRPPPK<br>LAAAILGGVDQIHIKPGAK<br>TNIIPVIEDAR<br>VSISEGDDKIEYR<br>IVALNAHTFLR<br>NLVPGESVYGEK<br>NLVPGESVYGEKR<br>NGGHFVISIK<br>AWNPFR
TMPO	Chromatin interaction	12.24	2	SSTPLPTISSSAENTR<br>YGVNPGPIVGTTR

B

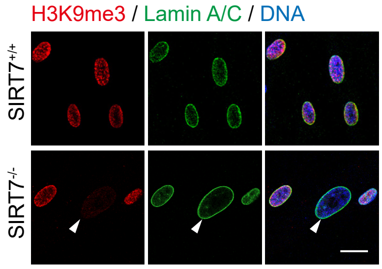

C

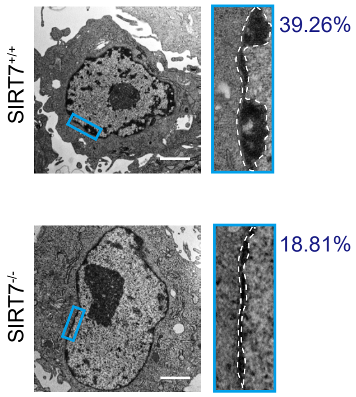

Figure S4

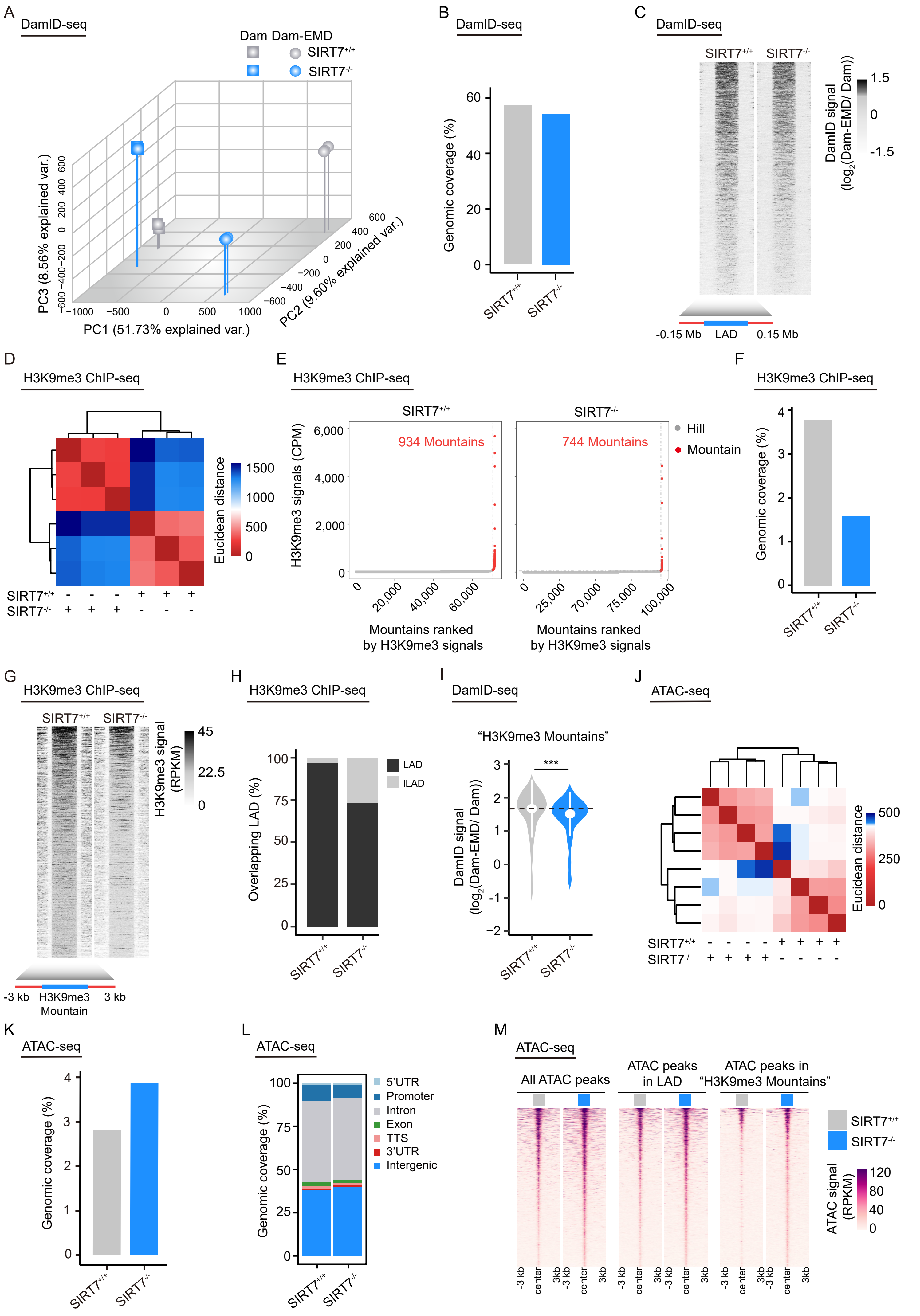
A
DamID-seq
Dam Dam-EMD
SIRT7+/+
SIRT7-/-
PC1 (51.73% explained var.)
PC2 (9.60% explained var.)
PC3 (8.56% explained var.)
B
DamID-seq
Genomic coverage (%)
SIRT7+/+
SIRT7-/-
C
DamID-seq
SIRT7+/+
SIRT7-/-
DamID signal (log2(Dam-EMD/ Dam))
1.5
0
-1.5
-0.15 Mb
LAD
0.15 Mb
D
H3K9me3 ChIP-seq
Eucidean distance
1500
1000
500
0
SIRT7+/+
SIRT7-/-
E
H3K9me3 ChIP-seq
SIRT7+/+
SIRT7-/-
934 Mountains
744 Mountains
Hill
Mountain
H3K9me3 signals (CPM)
Mountains ranked by H3K9me3 signals
F
H3K9me3 ChIP-seq
Genomic coverage (%)
SIRT7+/+
SIRT7-/-
G
H3K9me3 ChIP-seq
SIRT7+/+
SIRT7-/-
H3K9me3 signal (RPKM)
45
22.5
0
-3 kb
H3K9me3 Mountain
3 kb
H
H3K9me3 ChIP-seq
Overlapping LAD (%)
LAD
iLAD
SIRT7+/+
SIRT7-/-
I
DamID-seq
"H3K9me3 Mountains"
***
DamID signal (log2(Dam-EMD/ Dam))
SIRT7+/+
SIRT7-/-
J
ATAC-seq
Eucidean distance
500
250
0
SIRT7+/+
SIRT7-/-
K
ATAC-seq
Genomic coverage (%)
SIRT7+/+
SIRT7-/-
L
ATAC-seq
Genomic coverage (%)
5'UTR
Promoter
Intron
Exon
TTS
3'UTR
Intergenic
SIRT7+/+
SIRT7-/-
M
ATAC-seq
All ATAC peaks
ATAC peaks in LAD
ATAC peaks in "H3K9me3 Mountains"
SIRT7+/+
SIRT7-/-
ATAC signal (RPKM)
120
80
40
0
-3 kb
center
3kb

Figure S5

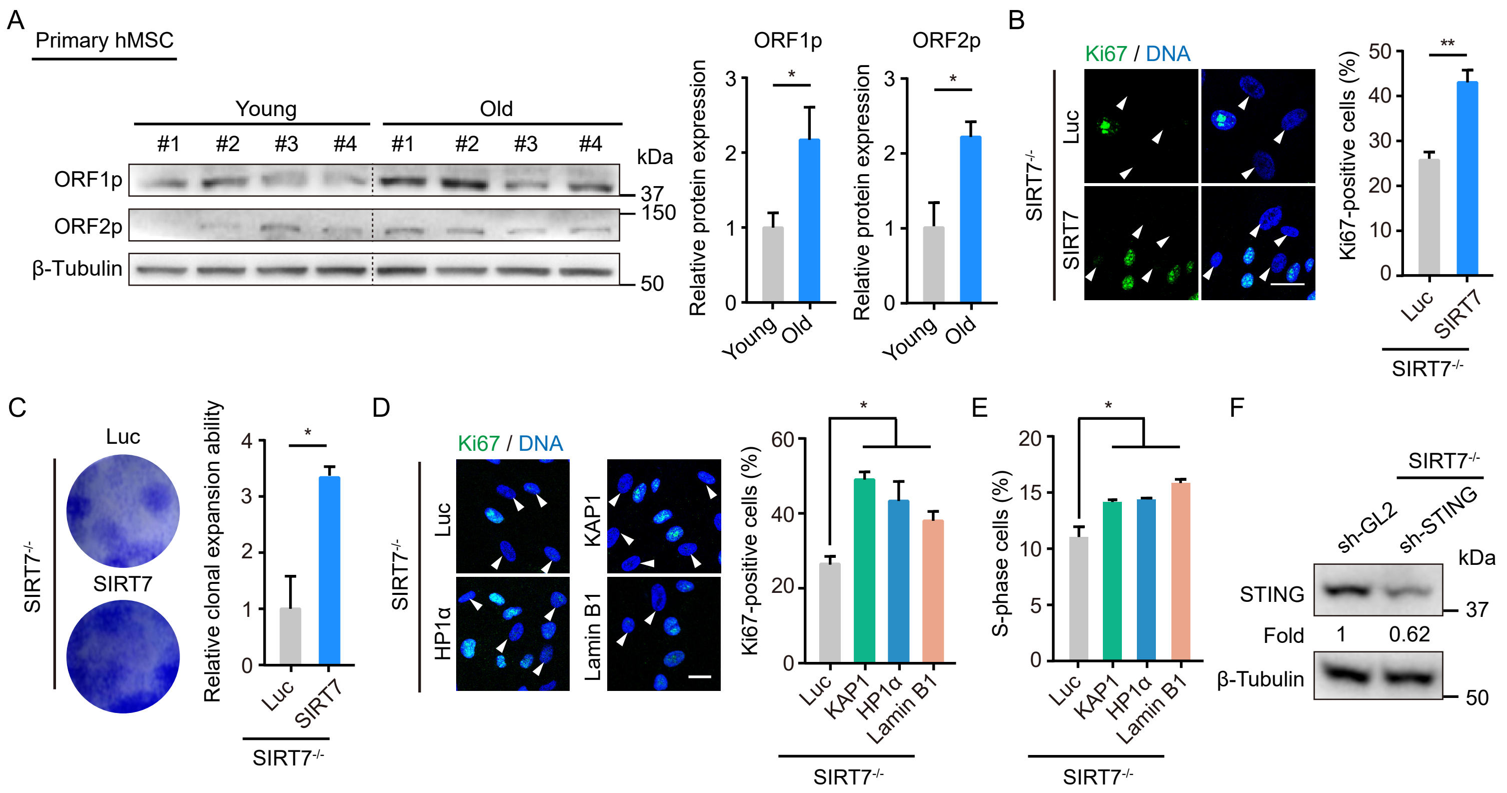
A
Primary hMSC
Young
Old
#1
#2
#3
#4
kDa
ORF1p
ORF2p
β-Tubulin
37
150
50
ORF1p
ORF2p
Relative protein expression
Young
Old
B
Ki67 / DNA
SIRT7-/-
Luc
SIRT7
Ki67-positive cells (%)
C
SIRT7
Relative clonal expansion ability
D
KAP1
HP1α
Lamin B1
E
S-phase cells (%)
F
sh-GL2
sh-STING
STING
Fold
1
0.62
